# The *Streptomyces leeuwenhoekii* genome: *de novo* sequencing and assembly in single contigs of the chromosome, circular plasmid pSLE1 and linear plasmid pSLE2

**DOI:** 10.1186/s12864-015-1652-8

**Published:** 2015-06-30

**Authors:** Juan Pablo Gomez-Escribano, Jean Franco Castro, Valeria Razmilic, Govind Chandra, Barbara Andrews, Juan A. Asenjo, Mervyn J. Bibb

**Affiliations:** Department of Molecular Microbiology, John Innes Centre, Norwich Research Park, Norwich, NR4 7UH United Kingdom; Centre for Biotechnology and Bioengineering (CeBiB), Department of Chemical Engineering and Biotechnology, Universidad de Chile, Beauchef 850, Santiago, Chile

**Keywords:** Second/Third next generation sequencing, Illumina MiSeq, Pacific Biosciences PacBio SMRT, Chaxamycin, Chaxalactin, Lasso peptide, Genome mining

## Abstract

**Background:**

Next Generation DNA Sequencing (NGS) and genome mining of actinomycetes and other microorganisms is currently one of the most promising strategies for the discovery of novel bioactive natural products, potentially revealing novel chemistry and enzymology involved in their biosynthesis. This approach also allows rapid insights into the biosynthetic potential of microorganisms isolated from unexploited habitats and ecosystems, which in many cases may prove difficult to culture and manipulate in the laboratory. *Streptomyces leeuwenhoekii* (formerly *Streptomyces* sp. strain C34) was isolated from the hyper-arid high-altitude Atacama Desert in Chile and shown to produce novel polyketide antibiotics.

**Results:**

Here we present the *de novo* sequencing of the *S. leeuwenhoekii* linear chromosome (8 Mb) and two extrachromosomal replicons, the circular pSLE1 (86 kb) and the linear pSLE2 (132 kb), all in single contigs, obtained by combining Pacific Biosciences SMRT (PacBio) and Illumina MiSeq technologies. We identified the biosynthetic gene clusters for chaxamycin, chaxalactin, hygromycin A and desferrioxamine E, metabolites all previously shown to be produced by this strain (*J Nat Prod*, 2011, 74:1965) and an additional 31 putative gene clusters for specialised metabolites. As well as gene clusters for polyketides and non-ribosomal peptides, we also identified three gene clusters encoding novel lasso-peptides.

**Conclusions:**

The *S. leeuwenhoekii* genome contains 35 gene clusters apparently encoding the biosynthesis of specialised metabolites, most of them completely novel and uncharacterised. This project has served to evaluate the current state of NGS for efficient and effective genome mining of high GC actinomycetes. The PacBio technology now permits the assembly of actinomycete replicons into single contigs with >99 % accuracy. The assembled Illumina sequence permitted not only the correction of omissions found in GC homopolymers in the PacBio assembly (exacerbated by the high GC content of actinomycete DNA) but it also allowed us to obtain the sequences of the termini of the chromosome and of a linear plasmid that were not assembled by PacBio. We propose an experimental pipeline that uses the Illumina assembled contigs, in addition to just the reads, to complement the current limitations of the PacBio sequencing technology and assembly software.

**Electronic supplementary material:**

The online version of this article (doi:10.1186/s12864-015-1652-8) contains supplementary material, which is available to authorized users.

## Background

Actinomycetes are Gram-positive mycelial bacteria found predominantly in soil, but they also occur in symbiotic associations with terrestrial and aquatic invertebrates. They undergo a complex process of morphological and physiological differentiation that leads to the production of exospores and specialised metabolites [1] with a wide range of biological activities. While the function of many of these molecules in the natural environment is not always evident, they are believed to provide a competitive advantage to the producing organism [2]. Many of these specialised metabolites possess potent antibiotic activity, and actinomycetes produce over 70 % of the natural product scaffolds found in clinically used anti-infective agents [3].

The pioneering sequencing and analysis of the genomes of *Streptomyces coelicolor* A3(2) [4] and *Streptomyces avermitilis* [5] revealed that actinomycetes possess an unexpected abundance of natural product biosynthetic gene clusters and thus that they have the potential to make many more compounds than previously thought. This led to the “genome mining” approach to natural product discovery, where bioinformatic analysis is used to estimate the biosynthetic capacity, and potential metabolite novelty, of an organism before extensive analysis in the laboratory. Genome mining of actinomycetes and other microorganisms has already provided access to many novel biosynthetic pathways and metabolites that otherwise would have remained undetected [6, 7]. It may be particularly useful when analysing novel and possibly difficult to culture microorganisms isolated from unusual and unexploited habitats such as the oceans, deserts, and the surfaces of plants and animals.

To be carried out efficiently, genome mining relies on the availability of a good quality genome sequence obtained at an affordable price and in a short time-frame. Until recently, whole genome shotgun sequencing using Next-Generation Sequencing (NGS) technologies could not be expected to yield less than several hundred contigs for an actinomycete genome of 6 – 12 Mb, with biosynthetic gene clusters often split over several contigs. Much bioinformatic analysis and contig-stitching by, for example, PCR would often be required to identify a complete cluster. Moreover, the short read length of Second Generation Sequencing technologies (like Illumina) makes it very difficult to correctly assemble the long repetitive sequences typically found in biosynthetic gene clusters containing modular polyketide synthases (PKSs) or non-ribosomal peptide synthetases (NRPSs). In such clusters, it is not unusual to find regions of high homology between genes and intragenic tandem repeats of 650-1000 bp with close to 100 % nucleotide sequence identity, well beyond the read length provided by Illumina (up to 2 × 300 bases); e.g., the *S. coelicolor* coelimycin PKS gene *sco6274*, positions 3879-4533 and 11986- 12639; and the calcium-dependent antibiotic NRPS gene *sco3230*, positions 13187-14121 and 16307-17241. The long sequence reads provided by Third Generation Sequencing techniques such as the Pacific Biosciences SMRT technology should allow more reliable assemblies of PKS and NRPS gene clusters and, in principle, yield single contigs for all of the replicons present in an actinomycete. The SMRT technology also produces more even sequence coverage of the high mol% G + C DNA found in actinomycete genomes than other NGS platforms [8].

*Streptomyces leeuwenhoekii* (formerly *Streptomyces* sp. C34) was isolated, together with many other novel actinomycetes, from the saline Chaxa Lagoon in the high-altitude Atacama Desert in northern Chile [9, 10]. It produces previously described metabolites (the siderophore desferioxamine E and the antibiotic hygromycin A), but also novel polyketide antibiotics, the chaxamycins and chaxalactins. Chaxamycin A – D are four novel ansamycin-type polyketides with promising antibacterial activity against MRSA and anti-proliferative activity resulting from the inhibition of the ATPase activity of the human Hsp90 protein [11, 12]. A draft genome sequence of this strain with 658 contigs had been derived previously from Illumina GA IIx 100 bp paired-end reads [10]; attempts to mine this sequence for the chaxamycin and chaxalactin biosynthetic gene clusters (Castro *et al.*, submitted) revealed many inconsistencies and misassemblies (see Additional file 1), presumably a consequence of the short read lengths of the Illumina technology and the repetitive nature of the two gene clusters.

Given the unusual origin of *S. leeuwenhoekii* and our desire to analyse its biosynthetic potential, we set out to sequence its genome using the most advanced technology available. This has allowed us to generate an almost complete chromosome sequence as well as the sequences of two plasmids as single contigs without recourse to gap-closing or sequencing of clones from a genomic library; to our knowledge, this is the first time that this has been achieved with an actinomycete. We also report here our findings on the advantages and limitations of the two technologies we used, Illumina MiSeq and Pacific Biosciences RSII, and we propose a pipeline for the generation of high quality actinomycete genome sequences.

## Results and Discussion

### Availability of data

The fully annotated sequences presented in this work have been deposited in the European Nucleotide Archive under Study accession number PRJEB8583 (http://www.ebi.ac.uk/ena/data/view/PRJEB8583). The sequences of pSLE1, pSLE2 and the chromosome have been assigned accession numbers LN831788, LN831789 and LN831790 respectively.

### Sequencing and assembly of the *S. leeuwenhoekii* genome

Two technologies were used to sequence genomic DNA isolated from *S. leeuwenhoekii*: Illumina MiSeq (as available in August 2013) and Pacific Biosciences (PacBio) RSII (as available in November 2013). Assembly of the Illumina sequencing data yielded 279 contigs, assembled into 175 scaffolds, totalling 8064420 bp. Assembly of the PacBio sequencing data produced three contigs of 7895833, 9613 and 94746 bp, totalling 8000192 bp. The PacBio contig of 7.9 Mb was expected to contain most of the sequence of the chromosome, and was referred to as C34-chromosome version 1. The small 9613 bp contig from the PacBio assembly was found to match the 7.9 Mb contig from position 5938858 to 5929122 (reverse complement) but with only 92 % identity, possibly indicating that it originated from reads with accumulated errors. These small, error-prone, contigs have been observed by others using PacBio sequencing (Silke Alt, Natalia Miguel-Vior, Zhiwei Qin and Thomas Scott, personal communications). This contig was discarded from any further analysis. A detailed description of the sequencing and bioinformatic analysis of this and following sections can be found in Additional file 2 – Materials and Methods.

### Correction of the PacBio 7.9 Mb contig (C34-chromosome version 1) using the Illumina assembly

Comparison of the Illumina MiSeq and PacBio assemblies readily identified problems of misassembly in the Illumina contigs, and in particular in the regions containing polyketide biosynthetic gene clusters, a particular focus of our initial interest in *S. leeuwenhoekii* (see Additional file 1: Figure S3). However, aware of the higher nucleotide-accuracy of the Illumina technology compared to PacBio, we mapped the Illumina MiSeq contigs (not scaffolds) over the PacBio-generated C34-chromosome version 1. The generated alignment was manually edited with GAP5 [13] to correct the PacBio sequence with the Illumina contigs while accommodating possible misassembles and systematic errors inherent in the Illumina technology [14]. Most of the differences were apparent omissions in the PacBio sequence of a C or G in homopolymeric runs of three or more Cs or Gs. These omissions were confirmed by analysis of individual sequence differences; the missing nucleotides in the PacBio sequence resulted in frame shifts that were readily identified using GC-Frame Plot [15] in Artemis [16] and substantiated by inspection of the corresponding amino acid sequences. Our analysis indicated that the Illumina sequence always contained the correct number of bases in these homopolymeric stretches, and consequently it was used to correct the PacBio assembly. This resulted in the insertion of 2934 bases and an additional 42 base changes, a total of 2976 corrections in a final chromosomal assembly of 7898767 bases (0.03768 %). This sequence was referred to as C34-chromosome version 2.

### Extension of 7.9 Mb corrected-contig (C34-chromosome version 2) with the Illumina assembly

During the previous correction step we identified sequence in the Illumina assembly that was absent from the PacBio assembly. This was achieved by using all of the Illumina assembled data instead of the Illumina reads, which is the usual bioinformatic practice (in e.g., software like iCORN [17] and Mira [18]). We found that Illumina contig 0089 (29048 bp) mapped at the 5’-end of the PacBio 7.9 Mb contig but contained 5.1 kb of additional sequence (see Additional file 3: Figure S3). After careful examination of the genetic content of contig 0089, we concluded that the extra 5.1 kb was genuine *S. leeuwenhoekii* sequence likely located at the end of the terminal inverted repeat (see below). BLAST analysis [19] and alignment in GAP5 were then used to extend the 5’-end of the chromosomal sequence with an extra 5121 bases to yield a chromosome of 7903888 bp, referred to as C34-chromosome version 3.

### Further correction of C34-chromosome version 3 with the Illumina paired-end reads

The original, unassembled, Illumina paired-end reads with quality values were aligned to version 3 of the chromosome with two different programs, BWA [20, 21] and Bowtie 2 [22], processed with SAMtools and bcftools [23] to call the potential variants, and these were then studied within GAP5. We focused on the additions/omissions reported by both alignment programs. While we decided not to incorporate any of the reported base changes, we did correct a few additions/omissions, in particular an erroneous addition at position 7169599 that was only reported by the BWA alignment (GTGGA was corrected to GTGA) which repaired a reading-frame shift in a polyketide synthase gene that is part of the chaxalactin biosynthetic gene cluster (Castro *et al.*, manuscript in preparation). Three corrections were also made at the beginning of the proposed Terminal Inverted Repeat (TIR) at the 5’-end of the sequence; a C was added in a run of five Cs after the G at position 387475, giving six Cs; a C was added after A at new position 387818 (acccaa to aCcccaa); and a C was added after A at new position 387824 (aacccca to aaCcccca). Finally, a G was added at position 7383828 (cggg to cGggg), correcting an omission reported by both programs that introduced a frame shift in an uncharacterised polyketide synthase gene (cluster 28 of Table 1).

**Table 1:** Putative biosynthetic gene clusters for specialised metabolites

antiSMASH Cluster No.	antiSMASH type descriptor	Position (*manually annotated clusters in italics*)		Our annotation (based on Ref.)
		From	To	
1	T1pks	99264	143430	
	*Not identified*	*160425*	*189028*	Hygromycin A [11, 44]
2	T1pks	191701	240196	
3	T1pks-nrps	324784	392261	
4	Nrps	379508	426758	
5	T3pks	416888	458084	
6	Bacteriocin	572464	582679	
7	Terpene	598795	619823	
	*Not identified*	*684373*	*654830*	Lasso-peptide 2
8	Nrps	714060	794426	
9	Terpene	1056004	1076960	
10	T2pks-transatpks-nrps	1075399	1155931	Halogenated polyketide [Razmilic et al.]
11	*T1pks-terpene*	*1211049*	*1289829*	Chaxamycin [Castro et al.]
12	T1pks	1497127	1544539	
13	Terpene	1624097	1645110	
14	T1pks-siderophore	1776281	1833813	
15	Terpene	1972277	1994487	
16	Bacteriocin	2013690	2025087	
17	Siderophore	2293580	2305424	Highly conserved
18	Nrps-t1pks	2668194	2719415	
19	T3pks	2937137	2978264	
20	*Terpene*	*3056325*	*3058819*	Albaflavenone [45, 46]
	Not identified	3560196	3564842	Lasso-peptide 1
21	*Siderophore*	*5237176*	*5244356*	Desferrioxamine E [11, 47, 48]
22	Melanin	5330379	5340933	
23	Amglyccycl-butyrolactone	5385171	5417416	
24	Ectoine	6176293	6186691	
25	Other	6710095	6751819	
26	T3pks	6822979	6864043	
27	T1pks	7141058	7240871	Chaxalactin [11, Castro et al.]
28	T1pks	7355977	7439461	
29	Other	7486047	7529121	
30	Terpene-t2pks	7530162	7588405	
31	Terpene	7744176	7768730	
	*Not identified*	*pSLE2 103389*	*pSLE2 105999*	Lasso-peptide 3

The final chromosomal sequence, referred to as C34-chromosome version 4, contains 7903895 nucleotides and has a mol% G + C content of 72.76 %.

### Annotation of the *Streptomyces leeuwenhoekii* chromosome

Annotation of the *S. leeuwenhoekii* chromosome version 4 was performed using Prodigal [24] to identify protein coding sequences (PCS) followed by BASys [25] for assignment of putative function. We chose gene identifiers starting with “sle”, for “*Streptomyces leeuwenhoekii*”, followed by five digits starting with “sle_00010” with increments of 10 to allow for the addition of subsequently identified genes and genetic features. Neither Prodigal nor BASys annotated rRNA or tRNA genes, so the chromosome sequence was submitted to the RAST server [26, 27] and the rRNA and tRNA annotations added to generate the published *S. leeuwenhoekii* chromosome sequence.

### Identification of the possible Terminal Inverted Repeats (TIR) and chromosome ends

*Streptomyces* species frequently possess linear chromosomes with Terminal Inverted Repeats (TIRs) of almost identical sequence with covalently-bound terminal proteins for priming of replication [28]. The length of the TIR is very variable among species, ranging from only 14 bp in *Streptomyces hygroscopicus* 5008 [29] to over 1 Mbp in *S. coelicolor* [30].

BLAST analysis and examination in Artemis revealed that the last 6996 bases at the 3’-end of the chromosome sequence (hereinafter referred to as “right TIR”) were 99 % identical to a segment of reverse-complementary sequence around position 388 kb (hereinafter referred to as “left TIR”; see Additional file 3, Fig. 1). It is likely that this 7 kb sequence at the right end represents the start of the right TIR, and was acquired by some of the long PacBio reads that extended from the region next to the TIR, while the rest of the reads from the right TIR have probably been assembled with the left TIR. Visualisation of the PacBio assembly BAM file revealed a pronounced increase in coverage of the first 380 kb of the assembly (see Additional file 3: Figure S2), probably reflecting the incorporation of reads from both the left and right TIR in the assembly of the left TIR.

**Fig. 1:**
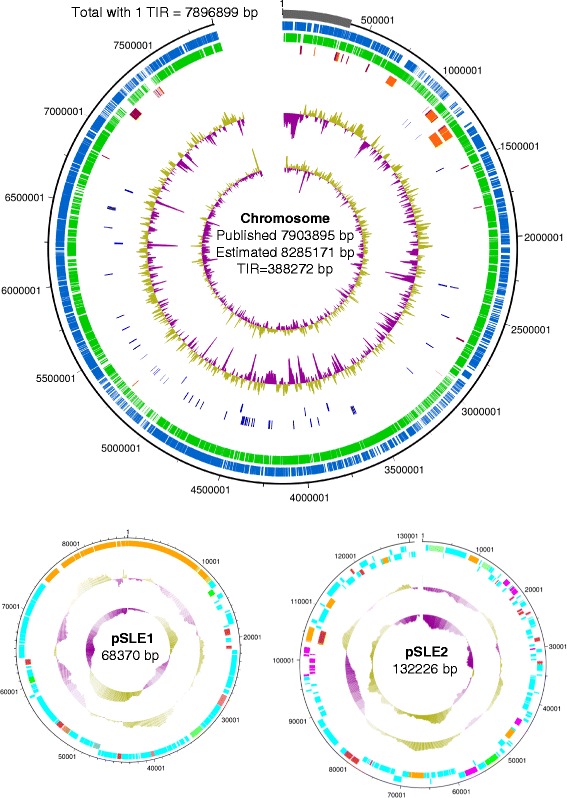
Schematic representation of the *S. leeuwenhoekii* chromosome, circular plasmid pSLE1, and linear plasmid pSLE2 (incomplete sequence). The chromosome is represented as an open circle, covering only the published sequence without the duplication of the terminal inverted repeat (represented as a grey band starting at position 1). From outside to inside, the concentric circles represent: nucleotide position; Protein Coding Sequences (PCSs) on the forward strand; PCSs on the reverse strand; PCSs for putative biosynthetic genes for specialised metabolites (dark red indicates the forward strand, orange the reverse strand); the orange box shown in the fifth circle indicates the chaxamycin biosynthetic gene cluster; tRNA and rRNA genes are shown in the sixth and seventh lines, respectively, in dark blue; the eighth concentric circle shows the GC-plot (GC %, window size = 10000; base step size = 200) and the inner-most circle the GC-skew ([(G − C)/(G + C)] window size = 10000; base step size = 200), both calculated using the sequence with both TIRs, a window size of 10000 and a step size of 200 (purple and olive indicate below and above average, respectively). For pSLE1 and pSLE2, PCSs are coloured red for putative regulatory genes; green, for plasmid replication and partitioning genes; the fourth circle in shows the GC-plot and the inner-most circle the GC-skew, both calculated as for the chromosome. For pSLE1, phage-related genes are shown in orange, and the type III PKS (chalcone synthase) gene is shown in brown. For pSLE2, genes with known plasmid functions are in orange; genes annotated as mobile elements and involved in transposition are in pink; the lasso-peptide biosynthetic gene cluster is shown in dark orange. Not to scale

**Fig. 2:**
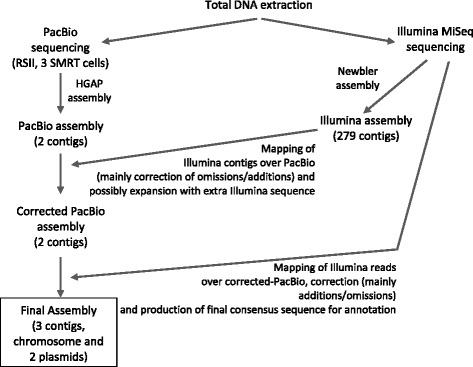
Sequencing and assembly pipeline. The sequencing and assembly pipeline followed in this work (data specific to this project are shown in brackets) and suggested as strategy for actinomycete genome sequencing

Detailed analysis of the end of the left TIR (the start of C34-chromosome version 4) identified two genes, *sle_00020* and *sle_00120*, with 84 % nucleotide sequence identity to each other, and predicted amino acid sequences that were 73 % and 72 % identical, respectively, to a helicase-like protein encoded by *sco0002* of *S. coelicolor*, homologues of which are present and highly conserved at both ends of all sequenced linear replicons (chromosomes and plasmids) from *Streptomyces* species [31, 32]. The presence of two contiguous helicase-like encoding genes has not been described before but detailed sequence inspection failed to reveal possible misassembly. Mfold [33] analysis of the DNA sequence upstream of *sle_00020* (but not *sle_00120*) revealed palindromic repeats with the potential to form a complex secondary structure (see Additional file 3: Figure S3) similar to those found in other *Streptomyces* linear replicons [34].

Our analysis suggests that the TIRs of the *S. leeuwenhoekii* chromosome extend for about 388 kb, at the upper end of published *Streptomyces* TIR sequences. This also implies that the hygromycin A biosynthetic gene cluster, located between position 160-189 kb, could be duplicated at each end of the chromosome should both TIRs show complete gene conservation. While still infrequent, biosynthetic gene clusters for specialised metabolites localised in the TIRs of actinomycetes have been described previously [35, 36] and demonstrated experimentally [35].

### Assembly and annotation of pSLE1, an 86.4 kb circular plasmid

The 94746 bp PacBio-contig contained direct repeats of 8.4 kb present at both ends, indicative of an 86 kb circular DNA molecule. Most of this sequence was also found in a contig of 86370 bp in the Illumina assembly. BLAST analysis using the non-redundant NCBI database suggested that it represents a plasmid, which we called pSLE1.

A detailed description of the assembly of the circular DNA sequence using both PacBio and Illumina data is given in the Additional file 2 and Additional file 4. pSLE1 possesses a base composition of 69 % mol% G + C. 133 putative PCSs were identified with Prodigal and tagged as “sle2_001”; putative functions were added manually in Artemis using BLAST and Pfam [37] searches.

For most of the PCSs, in particular those encoding putative phage particle proteins, the annotation was based on that of pZL12 [38], a well characterised plasmid with high levels of homology and synteny to pSLE1. Genes encoding ParA and ParB partitioning proteins were identified with Pfam, supporting the proposition that this contig represents an autonomously replicating element. pSLE1 ParA shows no discernible identity to the chromosomal ParA, but is 62 % identical to pZL12.17c ParA and 67 % identical to the putative plasmid partitioning protein ParA from *Streptomyces rochei* (sequence ID BAK19858). pSLE1 ParB shares 31 % identity with the chromosomal ParB, but 41 % identity with pZL12.16c ParB and 67 % identity with the putative plasmid partitioning protein ParB from *S. rochei* (sequence ID BAK19859). Interestingly, pSLE1 contains two genes encoding putative integrases, suggesting that the plasmid might be capable of insertion into the chromosome.

A search with antiSMASH [39] did not identify any putative specialised metabolite gene clusters in pSLE1 (a gene encoding a putative Type III PKS with 59 % amino acid sequence identity to pZL12-100 and similar to RppA from *Streptomyces antibioticus* was identified but, as in pZL12, the neighbouring genes did not indicate that it was part of a natural product gene cluster).

### Assembly and annotation of pSLE2, a >132 kb linear plasmid

Two Illumina contigs, contig0026 with 97005 bp and contig0079 with 32697 bp, did not match any sequence in the PacBio assembly. BLAST searches against the non-redundant NCBI database indicated that it could represent a plasmid, which we designated pSLE2. We were subsequently able to join the Illumina contigs using corrected PacBio reads to yield a 132 kb sequence (see Additional file 2 and Additional file 5 for details).

pSLE2 has a base composition of 70 mol% G + C. PCSs were identified using Prodigal and tagged as “sle2_001”; putative functions were manually annotated in Artemis using BLAST and Pfam searches.

We could not identify a clear direct repeat indicative of a circular plasmid, as we did for pSLE1, or a putative TIR characteristic of a linear plasmid, suggesting that some sequence might be missing. However, the 5’-end of pSLE2 (provided by the 5’-end of Illumina contig 0026) is highly similar (over 85 % nucleotide identity) to the end of the TIR identified for the chromosome (see Additional file 5: Figure S2), and includes a gene for a putative terminal helicase (*sle2_002*) with 87 % and 85 % amino acid identity to Sle_00020 and Sle_00120, respectively. Such high identity between linear chromosomes and linear plasmids is a feature of *Streptomyces* species [31] and suggests that pSLE2 is a linear plasmid and that this sequence is at one end of a TIR.

Two contiguous genes were found by Pfam searches to encode possible functional homologues of the partition proteins ParA and ParB (no significant identity was found to any of the putative partitioning proteins encoded by the chromosome or pSLE1, further suggesting that this is a separate replicon). Pulse-Field Gel-Electrophoresis (PFGE) of total DNA isolated from *S. leeuwenhoekii* revealed an extrachromosomal replicon with an apparent linear size of between 112 and 130.5 kb (Additional file 5: Figure S1), consistent with the predicted size of pSLE2.

As for pSLE1, we found a gene, *sle2_153*, encoding a putative integrase, suggesting that the plasmid might be capable of integrating into the chromosome. In addition, we identified at least three genes encoding possible conjugation functions (*sle2_062*, *sle2_090* and *sle2_091*) suggesting that the plasmid might be capable of self-transmission by conjugation.

An interesting feature is the presence of a gene, *sle2_140*, encoding a putative zeta-toxin (Pfam family “Zeta_toxin” (PF06414) e-value 1.9e-45) potentially involved in ensuring the maintenance and segregation of the plasmid [40]; we did not find a gene encoding a putative anti-toxin, but we did find a toxin-antitoxin system encoded in the chromosome similar to that reported for *S. coelicolor* and *Streptomyces lividans* [41].

AntiSMASH did not reveal any putative natural product gene clusters, but we did find a gene cluster potentially encoding the biosynthesis of a lasso-peptide, which we have subsequently identified and characterised (manuscript in preparation).

Intriguingly, there are several genes encoding putative transposases, many with frame shifts (revealed using GC Frame Plot). This region was not present in the Illumina assembly, and lies between Illumina contigs 0026 and 0079; only the PacBio corrected-reads contained the full sequence (see Additional file 2). The presence of many repeated sequences with high levels of nucleotide sequence identity may explain their absence from the Illumina assembly.

### General characteristics of the genome sequence

Our assembled chromosome sequence contains 7903895 bp, with a mol% G + C content of 73 %, consistent with other members of the genus *Streptomyces*. With the proposed addition of the right hand 388 kb TIR, the predicted size of the genome would be 8285171 bp (our 7903895 bp assembly, minus 6996 bp of the start of right TIR, plus the duplication of the left TIR of 388272 bp, equals 8285171 bp for the chromosome with two equal 388 kb TIRs). This is similar to the size of many streptomycete genomes (e.g., the chromosome of *S. coelicolor* is 8667507 bp [1]).

6712 PCSs were predicted for our assembled sequence, and 7057 PCSs if we include the PCSs predicted for the right TIR. A table of COG (Clusters of Orthologous Genes) functional categories [42] was determined (Table 2).

**Table 2:** COG functional categories. COG (Clusters of Orthologous Genes) functional categories of chromosomal protein codding sequences identified in S. leeuwenhoekii chromosome, and from S. coelicolor for comparison (as calculated by BASys [25] for both genomes)

	*Streptomyces leeuwenhoekii*		*Streptomyces coelicolor*	
COG functional categories	Percentage	Number	Percentage	Number
Energy production and conversion	4	270	4.1	317
Cell division and chromosome partitioning	0.5	30	0.4	31
Amino acid transport and metabolism	6	400	5.1	395
Nucleotide transport and metabolism	1.3	89	1.2	93
Carbohydrate transport and metabolism	6.5	433	6.5	503
Coenzyme metabolism	2.3	154	2.2	170
Lipid metabolism	3.7	250	3.3	255
Translation, ribosomal structure and biogenesis	2.7	182	2.5	193
Transcription	7.3	493	8.5	658
DNA replication, recombination and repair	2.9	193	2.9	224
Cell envelope biogenesis, outer membrane	3	204	2.9	224
Cell motility	0.1	5	0.1	8
Posttranslational modification, protein turnover, chaperones	1.9	127	1.8	139
Inorganic ion transport and metabolism	2.3	154	2.7	209
Secondary Structure	2.8	186	1.9	147
General function prediction only	6.9	460	7.4	572
COG of unknown function	3.6	243	3.6	278
Signal Transduction	3.8	256	4.2	325
Unknown	36.6	2458	36.7	2839

The *S. leeuwenhoekii* chromosome contains six rRNA operons located between nucleotide positions 2364598 and 6687647; interestingly, an orphan gene encoding a 5S rRNA was found at position 3962518. DnaA and DnaN replication proteins are encoded in genes spanning nucleotide positions 4260071-4258170 and 4256851-4255721, respectively, indicating that the likely origin of replication of the chromosome lies between 4256851 and 4258170. Consistent with precedent, the two rRNA operons that lie between nucleotide positions 2364598 and 3779582 (and the 5S rRNA orphan gene at position 3962518) are located on the complementary strand, while the four rRNA operons located between nucleotide positions 4473032 and 6687647 are located on the top strand (see Additional file 6: Table S1). We identified 65 tRNA genes (see Additional file 6: Table S2), similar to the number found in other *Streptomyces* (e.g., the *S. coelicolor* genome contains 64 tRNA genes and one tRNA pseudogene).

The general characteristics of the *S. leeuwenhoekii* genome are summarised in Table 3.

**Table 3:** General characteristics of the S. leeuwenhoekii genome

Assembled chromosome size	7903895 bp
Estimated chromosome size	8285171 bp
Estimated Terminal Inverted Repeats	388272 bp
Chromosome topology	Linear
Chromosome G + C content	73 %
rRNA operons	6
tRNA genes	65
pSLE1 circular plasmid	86370 bp
pSLE1 G + C content	69 %
pSLE2 linear plasmid	132226 bp
pSLE2 G + C content	70 %
Putative biosynthetic gene clusters for specialised metabolites	34 (+1 in pSLE2)

### Biosynthetic gene clusters for specialised metabolites

Putative gene clusters encoding the biosynthesis of specialised metabolites were identified with antiSMASH 2 [39] and non-automated searches with BLAST and Pfam (Table 1). *S. leeuwenhoekii* was known to produce the siderophore desferrioxamine E and the antibiotic hygromycin A [11]; we identified probable biosynthetic gene clusters for both of these metabolites by searching for homologues of the corresponding gene clusters from *S. coelicolor* and *Streptomyces hygroscopicus* NRRL 2388, respectively. The desferrioxamine E gene cluster extends from *sle_44550* to *sle_44600* and shows complete gene synteny with the homologous gene cluster from *S. coelicolor* with protein identities varying between 77 % and 93 %, while the putative hygromycin A gene cluster, which spans from *sle_01610* to *sle_01870*, shows almost complete synteny with the homologous gene cluster from *S. hygroscopicus*, with protein identities of 64 % to 90 %. Interestingly, antiSMASH did not identify the hygromycin A gene cluster which lies within the proposed left TIR; two putative type-I PKS gene clusters and one putative hybrid type-I PKS-NRPS gene cluster were also found in the left TIR. If, as we have proposed, the TIRs span for 388 kb, then all four of these gene clusters would be duplicated at the other end of the chromosome.

*S. leeuwenhoekii* is also known to produce two novel families of polyketide antibiotics, the chaxalactins and the ansamycin-like chaxamycins [11, 12]. The biosynthetic gene clusters for both antibiotics were identified and are currently under investigation.

We also identified three putative gene clusters each encoding the biosynthesis of unknown lasso-peptides (LS). Two of them, the “lasso-peptide 1” and “lasso-peptide 2” gene clusters (named *ls1* and *ls2*, respectively) are located on the chromosome, while the “lasso-peptide 3” gene cluster (named *ls3*) is located on the linear plasmid pSLE2. All three gene clusters are currently under study in our laboratory, where PCR amplification and Sanger sequencing have confirmed that the assembly and sequence reported here is correct. These clusters were not identified by antiSMASH.

## Conclusions

### Benefits and challenges of sequencing technologies: a revised pipeline

To our knowledge, this is the first report of the use of NGS to produce a high quality and non-fragmented genome sequence of an actinomycete, an essential prerequisite for efficient genome mining for natural product discovery in these GC-rich bacteria. Our assembly yielded the sequences of a single chromosomal contig, a complete circular plasmid (pSLE1), and most of a linear plasmid (pSLE2). Although the Illumina MiSeq assembly produced a single contig for pSLE1, it would not have been possible to confirm its circularity and completeness without the PacBio data. Overall, we used two SMRT cells (plus a small amount of data from a faulty run) resulting in over 120x coverage. Thus, with the improving capacity of the PacBio SMRT cells, we predict that a maximum of three SMRT cells should be sufficient to obtain the entire complement of replicons of an actinomycete genome of around 8 Mb in single contigs. This contrasts with the previously published Illumina draft genome sequence of *S. leeuwenhoekii* [10] that contained 658 contigs in which we found many misassemblies and missing sequence (see Additional file 1).

The PacBio long reads also permitted the identification of the start of the right hand TIR, but they did not provide any sequence information for the 5 kb at the ends of the chromosome and the linear plasmid pSLE2, which was obtained from the Illumina data. The reason for this is unknown, but it could reflect the different strategies used for library construction; while PacBio focuses on large fragments of ~20 kb, the average size of the Illumina library used for sequencing was 550 bp. Also, no specific procedure was used during DNA purification to remove the protein that is covalently bound to the termini of the linear replicons, perhaps resulting in lower sequence coverage of the terminal regions that might be further exacerbated by the large fragment size used for PacBio sequencing.

Surprisingly, the PacBio assembly lacked the linear pSLE2 sequence, although the sequence information was present in the corrected PacBio reads. The Illumina assembly contained two large contigs covering most of pSLE2 that were merged into a contiguous sequence using the PacBio corrected reads, but this plasmid would have not been identified with the PacBio assembly alone.

These two findings highlight the importance of the current need to use both PacBio and Illumina assemblies, instead of assembling only the PacBio data and then using the Illumina reads to correct the assembly, which appears to be the accepted practice [17, 18, 43]. We cannot explain the lack of assembly of some of the PacBio data present in the corrected reads (it might be due to a difference in relative abundance compared to the rest of the sequence, but this will require analysis of the PacBio assembly algorithm) but this was compensated for by using the Illumina MiSeq assembly.

In summary, for a *de novo* shotgun genome sequence from an actinomycete aimed at yielding single contigs per replicon, we currently propose a strategy (Fig. 2) that includes sequencing genomic DNA with PacBio RSII using initially two (and a third later if required) SMRT cells and a >20 kb insert library (aiming at >100x coverage) combined with Illumina MiSeq paired-end sequencing of a 500 bp library without PCR amplification (to avoid introducing bias from uneven amplification of high G + C actinomycete DNA (aiming at >90x coverage)). Both data sets are assembled and compared, and the Illumina contigs used to correct the PacBio nucleotide omissions/additions, which should be confirmed using GC Frame Plot and BLAST analyses. This consensus is further corrected with the Illumina reads. Despite the highly efficient current assembly algorithms, a considerable amount of human input was still needed to obtain a high quality single contig assembly, and accurate annotation of gene function.

## Methods

Extensive details of the methodology and materials used during this study are given in Additional file 2. Perl scripts are given in Additional file 7 and Additional file 8.

## Additional files

Additional file 1:
**Comparison of assemblies and examples of misassemblies (using the Artemis Comparison Tool).** Three figures illustrating the problems of assembly of polyketide synthase genes with Illumina data. Additional file 2:
**Materials and Methods.** This Microsoft Word file contains all methodology and materials used to extract and sequence the genomic DNA, and the software used, pipelines, strategies and decisions followed during bioinformatic analysis. A detailed description of the process followed to compare assemblies, map reads and correct the chromosome is included, as well as a detailed description of the process used to assemble the two plasmids. It also includes details of the process used for annotation of gene function. Additional file 3:
**Determination of Terminal Inverted Repeat.** Three figures illustrating and supporting the identification of the Terminal Inverted Repeat. Additional file 4:
**Assembly of circular plasmid pSLE1.** Figure illustrating the organisation of Illumina and PacBio data covering pSLE1. Additional file 5:
**Assembly of linear plasmid pSLE2.** Two figures showing an extrachromosomal replicon, possibly pSLE2, after Pulse Field Gel Electrophoresis, and the similarity between the chromosome and pSLE2 left ends. Additional file 6:
**rRNA operons and tRNA genes.** Two tables with the identified rRNA operons and tRNA genes. Additional file 7:
**Perl script “fastaToIndividualFiles.pl”.** Perl script (text file) used to extract from a multi-fasta file, and rename at the same time, specific sequences whose identifiers are listed in a text file (if a list is not provided, all sequences are output to individual files). Additional file 8:
**Perl script “renameSequencesInFasta.pl”.** Perl script (text file) used to extract from a multi-fasta file, and rename including the sequence length at the same time, specific sequences whose identifiers are listed in a text file (if a list is not provided, all of the sequences are output to individual files).
