# Supplementary material for: The Streptomyces leeuwenhoekii genome: de novo sequencing and assembly in single contigs of the chromosome, circular plasmid pSLE1 and linear plasmid pSLE2
Source: BMC Genomics. 2015 Jun 30;16(1):485. doi: 10.1186/s12864-015-1652-8 (PMC4487206; doi:10.1186/s12864-015-1652-8)

**The *Streptomyces* *leeuwenhoekii* genome: *de novo* sequencing and assembly in single contigs of the chromosome, circular plasmid pSLE1 and linear plasmid pSLE2.**

### Juan Pablo Gomez-Escribano^1*^, Jean Franco Castro^1,2^, Valeria Razmilic^1,2^, Govind Chandra^1^, Barbara Andrews^2^, Juan A. Asenjo^2^, Mervyn J. Bibb^1^

^1^Department of Molecular Microbiology, John Innes Centre, Norwich Research Park, Norwich, NR4 7UH, United Kingdom

^2^Centre for Biotechnology and Bioengineering (CeBiB), Universidad de Chile, Beauchef 850, Santiago, Chile

## Availability of data

The fully annotated sequences presented in this work have been deposited in the European Nucleotide Archive under Study accession number PRJEB8583 (<http://www.ebi.ac.uk/ena/data/view/PRJEB8583>). Each sequence has been assigned the accession codes:

**Replicon Accession ENA_Link**

pSLE1 LN831788 <http://www.ebi.ac.uk/ena/data/view/LN831788>

pSLE2 LN831789 <http://www.ebi.ac.uk/ena/data/view/LN831789>

Chromosome LN831790 <http://www.ebi.ac.uk/ena/data/view/LN831790>

**Additional File 1:**

**Comparison of assemblies and examples of misassemblies (using the Artemis Comparison Tool)**

## Additional File 1: Figure S1 – Example of misassembly in Busarakam *et al*.’s sequence.

Contig 642 of the sequence published by Busarakam and co-workers (top sequence) covers part of the chaxamycin biosynthetic gene cluster assembled by PacBio (bottom sequence). Genes for the biosynthesis of 3-amino-5-hydroxybenzoic acid (in red) have been correctly assembled, but the stretch of sequence covering the polyketide synthase genes *cxmE* and *cxmD* is wrongly assembled (in yellow); in addition, part of *cxmD* is missing from Busarakam’s sequence (the space between two yellow strips in the bottom sequence).


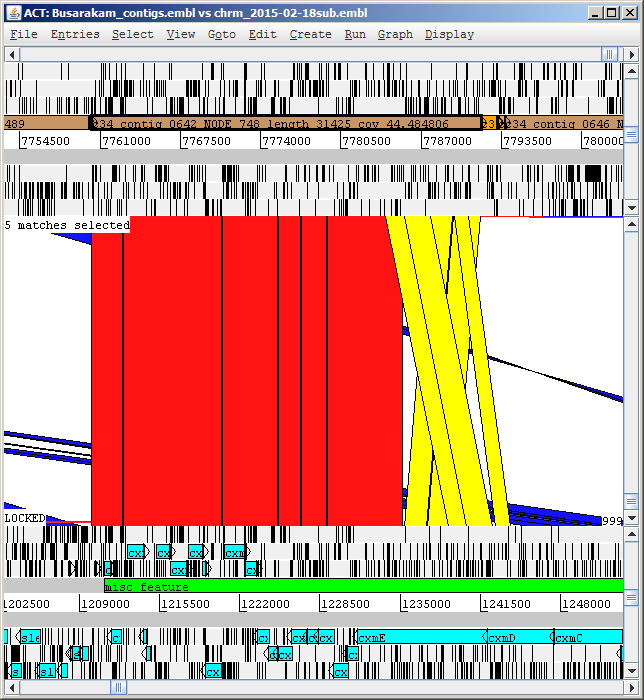


## Additional File 1: Figure S2 – Example of misassembly in Busarakam’s sequence.

Contig 615 of the sequence published by Busarakam and co-workers (top sequence) covers part of the chaxamycin biosynthetic gene cluster assembled by PacBio (bottom sequence). In this case, the contig contains most of the polyketide synthase gene *cxmB*, but with some gaps (the spaces between the red strips) and the neighbouring genes encoding part of the modular polyketide synthase have been misassembled (the yellow strips).


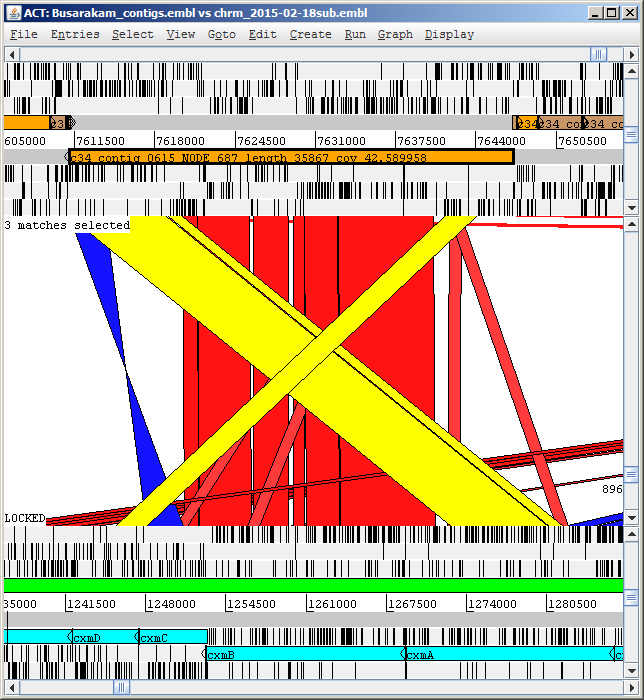


## Additional File 1: Figure S3 – Example of misassembly in the Illumina MiSeq contigs

Contig 0006 of the sequence obtained with Illumina MiSeq (top sequence) covers about half of the chaxamycin polyketide synthase genes (*cxmA* and *cxmB*) as assembled by PacBio (bottom sequence). However, the 3’-end of Contig 0006 seems to have been misassembled, with the highest identity (86%) being with the PKS gene *cxmE* (yellow strips). The Illumina assembly covering the PKS genes *cxmD* and *cxmE* is also problematic. The dark red colour indicates identity of at least 99%, and the less intense red colour indicates identity over 80%.


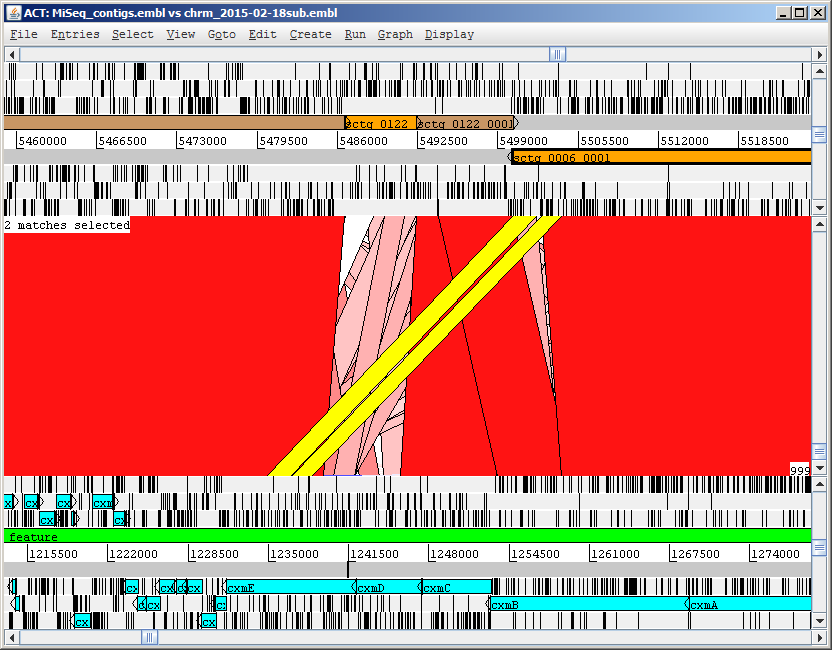

Supplement: Additional file 1: — Comparison of assemblies and examples of misassemblies (using the Artemis Comparison Tool). Three figures illustrating the problems of assembly of polyketide synthase genes with Illumina data. [file 12864_2015_1652_MOESM1_ESM.docx]
