# Supplementary material for: The Streptomyces leeuwenhoekii genome: de novo sequencing and assembly in single contigs of the chromosome, circular plasmid pSLE1 and linear plasmid pSLE2
Source: BMC Genomics. 2015 Jun 30;16(1):485. doi: 10.1186/s12864-015-1652-8 (PMC4487206; doi:10.1186/s12864-015-1652-8)
Supplement: Additional file 2: — Materials and Methods. This Microsoft Word file contains all methodology and materials used to extract and sequence the genomic DNA, and the software used, pipelines, strategies and decisions followed during bioinformatic analysis. A detailed description of the process followed to compare assemblies, map reads and correct the chromosome is included, as well as a detailed description of the process used to assemble the two plasmids. It also includes details of the process used for annotation of gene function. [file 12864_2015_1652_MOESM2_ESM.doc]

**The *Streptomyces* *leeuwenhoekii* genome: *de novo* sequencing and assembly in single contigs of the chromosome, circular plasmid pSLE1 and linear plasmid pSLE2.**

### Juan Pablo Gomez-Escribano^1*^, Jean Franco Castro^1,2^, Valeria Razmilic^1,2^, Govind Chandra^1^, Barbara Andrews^2^, Juan A. Asenjo^2^, Mervyn J. Bibb^1^

^1^Department of Molecular Microbiology, John Innes Centre, Norwich Research Park, Norwich, NR4 7UH, United Kingdom

^2^Centre for Biotechnology and Bioengineering (CeBiB), Universidad de Chile, Beauchef 850, Santiago, Chile

**Availability of data**

The fully annotated sequences presented in this work have been deposited in the European Nucleotide Archive under Study accession number PRJEB8583 (<http://www.ebi.ac.uk/ena/data/view/PRJEB8583>). Each sequence has been assigned the accession codes:

**Replicon Accession ENA_Link**

pSLE1 LN831788 <http://www.ebi.ac.uk/ena/data/view/LN831788>

pSLE2 LN831789 <http://www.ebi.ac.uk/ena/data/view/LN831789>

Chromosome LN831790 <http://www.ebi.ac.uk/ena/data/view/LN831790>

**Additional File 2:**

# Materials and Methods

## Preparation of high molecular-weight DNA

*Streptomyces leeuwenhoekii* type strain (C34^T^ = DSM 42122^T^ = NRRL B-24963^T^) was obtained from Michael Goodfellow and Alan Bull [1]. Petri plates with SFM (MS) agar medium [2] were inoculated with *S.* *leeuwenhoekii* and cultivated at 30 °C until full sporulation was observed (usually about one week); spores were prepared following the “*Streptomyces* spore suspension” protocol in Reference 2. Erlenmeyer Flasks (250 ml) with stainless-steel springs at the bottom and containing 50 ml of TSB::YEME 50::50 (recipes for both media from Reference 2) were inoculated with about 10e8 spores of *S. leeuwenhoekii* and cultivated at 30 °C for 48h (early stationary phase). The culture was divided into 5 ml aliquots and the mycelium harvested by centrifugation at 4000 r.p.m. for 5 min at room temperature; the supernatant was discarded, and the mycelium washed with 5 ml of 20% glycerol; aliquots not to be immediately processed were stored in 20% glycerol at -20 °C. Washed mycelium was harvested by centrifugation at 4000 r.p.m. for 5 min at room temperature, providing about 0.5 ml of wet mycelial pellet that was used as starting material for the salting-out protocol described in Reference 2, using the same volume of solutions despite the lower amount of starting mycelium; incubation times with lysozyme and proteinase K were increased as required until a clear lysate was obtained.

## Pulse-Field Gel-Electrophoresis (PFGE)

DNA was separated in a 1% agarose gel in 1x TBE buffer, using a CHEF DR II (Biorad) with 0.5x TBE running buffer continuously refrigerated at 14 °C , at 6 V/cm (current 110-125 mA) for 18 h. MidRange I PFG (New England Biolabs N3551S) was used as size marker. Visualisation was performed after staining with ethidium bromide after the electrophoresis, following standard methods [3].

## General visualisation, analysis, and manipulation of DNA sequence data

Visualisation and manipulation of text files was performed with advanced text editors Notepad++ (<http://notepad-plus-plus.org/>) or TextWrangler (<http://www.barebones.com/products/textwrangler/>) under Windows 7 64bits or MacOSX 10.6.8 respectively. DNA sequence analysis and annotation were performed with Artemis [4]. Visualisation of BAM alignment files was performed with BamView [5]. Comparison of assemblies and genomes was computed with WebACT ([http://www.webact.org](http://www.webact.org/)) and analysed with the Artemis Comparison Tool ACT [6]. BLAST+ [7] searches were performed at the NCBI web server (<http://www.ncbi.nlm.nih.gov/blast/>), at a John Innes Centre internal server, or on a standalone computer with prfectBLAST 2.0 [8] for less computing-intensive personalised databases. Pfam [9] searches were performed at the Pfam web server (<http://pfam.xfam.org/>). For automatic manipulation of multi-sequence fastA and fastaQ files, for extraction of individual sequences or renaming of sequences, Perl scripts were used (Additional Files 2 and 3) run on Perl 5, version 18, subversion 2 (v5.18.2) built for MSWin32-x64-multi-thread, installed on Windows 7 64bits as Strawberry Perl (5.18.2.2-64bit) package (http://strawberryperl.com). Additional Perl scripts used during this study are included in the NGS-TOOLBOX (<http://sourceforge.net/projects/ngs-toolbox/>) from David Rosenkranz, Institute of Anthropology, Johannes Gutenberg University Mainz, Germany (<http://www.anthropologie.uni-mainz.de/487_ENG_HTML.php>). Staden Package version 2.0.0b10 was used extensively for assembly and DNA sequence analysis [10,11]. Automated searches for natural product biosynthetic gene clusters were performed at the antiSMASH 2 server [12]. COG (Clusters of Orthologous Genes) functional categories [13] were calculated with BASys [14].

## DNA sequencing and assembly with Illumina MiSeq

Illumina MiSeq sequencing was carried out at the DNA sequencing facility in the Department of Biochemistry, University of Cambridge (80 Tennis Court Road, Cambridge, CB2 1GA, UK) in August 2013; sequencing was coordinated by Shilo Dickens and assembly performed by Markiyan Samborskyy. Details provided by the sequencing supplier follow. A shotgun paired-end library (average size of 550 bp) was prepared using the Illumina Nextera kit; no PCR amplification was performed. Sequencing was performed in an Illumina MiSeq device, running MiSeq Reporter version 2.2.29, with sequencing reagents MiSeq 500-cycle v2 chemistry. 89.71% of clusters passed the filter and 85.5% of bases qualified higher than Q30 at 2x250 nt. The 250 nt long paired-end reads were assembled with Newbler v2.9, using an Error Probability Cumulative (epc) filter of 0.1 in a 10 bp window. 1.24 Gb from 5.9 million reads were assembled in a total of 8087856 bases (calculated coverage 153x) in 387 contigs; of these, 279 contigs were larger than the established threshold of 500 nt, totalling 8064420 bases, the average contig was 28904, N50 contig size was 59284 and the largest contig was 157225 nt. The 279 contigs were further assembled into 175 scaffolds; for all of the analysis described in this work only the Illumina contigs were used, not the scaffolds.

## DNA sequencing and assembly with Pacific Biosciences

Pacific Biosciences (PacBio) sequencing was commissioned to The Genome Analysis Centre (Norwich Research Park, Norwich, NR4 7UH, UK; sequencing and assembly coordinated and performed by David Baker, Darren Heavens, Matt Clark and Chris Watkins) in November 2013. Details provided by the sequencing supplier follow. High molecular weight DNA was sheared with g-TUBE (Covaris PN 520079) aiming at DNA fragments of about 20 kb. The library was constructed with a 20kb size-selected protocol using DNA Template Prep Kit 2.0 (PN 001-540-726), purified and further selected for long insert size with a 0.35X AMPure (AMPure PB PN 100-265-900) bead size selection. The library was sequenced on a PacBio RSII device using the reagents DNA/Polymerase Binding Kit P4 (PN 100-236-500), DNA Sequencing Reagent 2.0 PN (100-216-400), SMRT®Cell V3 (PN 100-171-800) and Magbead (PN 100-133-600), loading at 200 pM on the plate. Data were collected with Stage Start with 180 minute movies. Three SMRT®Cell V3 cells were used yielding ~77 Mbp raw data with ~3.4 Kbp mean read length (the first cell underperformed for unknown reasons); ~525 Mbp raw data with ~5.8 Kbp mean read length; and ~425 Mbp raw data with ~5.4 Kbp mean read length; after quality filtering a combined total of ~966 Mbp with a ~5.4 Kb mean read length were obtained. Data were assembled with HGAP 2.0 [15] into 3 contigs of 7895833, 9613 and 94746 bases, totalling 8000192 bases (calculated coverage 121x). The 7.9 Mb contig is referred to as C34-chromosome version 1.

## Comparison of Illumina and PacBio assemblies

Both the Illumina and PacBio assemblies were compared using two approaches:

a. Using the Artemis Comparison Tool: WebAct (<http://www.webact.org/>) was used to compute the identity table and ACT [6] for visualisation of the Illumina contigs laid over PacBio contigs.

b. A BLAST [7] database was created with the PacBio assembly and queried with the Illumina contigs (not scaffolds) using Megablast [7] selecting a table output with only 4 hits (HSPs) per query sequence (ideally, each Illumina contig should give only one HSP covering the full contig, but this setting would allow for cases in which the Illumina contig is split over several HSPs). The table was analysed in a Microsoft Excel spreadsheet: HSPs under 97% identity and covering less than 1/4 of the query sequence were rejected; query sequences were sorted according to start position of subject (PacBio contig); although time consuming, this allowed us to identify Illumina contigs that did not match any PacBio assembled sequence, as well as stretches of PacBio sequence not covered by the Illumina data (probably because of mis-assembly of Illumina sequence reads).

## Mapping of Illumina contigs over PacBio 7.9 Mb contig and editing with GAP5

BWA [16,17] version 0.7.6a-r433 compiled (February 2014) and run on Mac OS X 10.6.8 (on a computer with a 2.93 GHz Intel Core i7 and 16 GB of 1333 MHz DDR3 RAM) was used to align all Illumina contigs over the largest PacBio contig of 7.9 Mb (which we considered to contain most of the chromosome of *S. leeuwenhoekii*). We used the “bwtsw” indexing algorithm and the “bwa-mem” alignment algorithm, both with the default options. The alignment output in SAM format was converted to BAM format, sorted and indexed with SAMtools [18] version 0.1.19-44428cd (compiled, on February 2014, and run as BWA). The sorted-BAM alignment was converted to a GAP5 database with “tg_index” program included in Staden Package version 2.0.0b10 [10,11]. The commands used were (arbitrary file names given in italics):

bwa index -p *database_prefix* -a bwtsw *PacBio_contig.fna*

bwa mem *database_prefix* *illumina_contigs.fna* > *alignment.sam*

samtools view -b -S -o *alignment.bam* *alignment.sam*

samtools sort *alignment.bam alignment-sort*

samtools index *alignment-sort.bam*

tg_index -b *alignment-sort.bam*

## Mapping of Illumina paired-end reads over C34-chromosome version 3 and editing with GAP5

Illumina paired-end reads with quality values were aligned to version 3 of the chromosome with two different programs, BWA and Bowtie 2.

BWA [16,17] version 0.6.1-r104 (Windows 64bits binary from <https://bow.codeplex.com/releases/view/86475>; run on Windows 7 64 bits on a computer with a 3.20 GHz Intel Core i5-4570 and 16 GB of RAM). We used the “is” default indexing algorithm and the “bwa-bwasw” alignment algorithm, both with the default options.

Bowtie [19] version 2.0.0.5 was run on the AMD64-based High Performance Computing cluster at the Norwich Biosciences Institutes, running CentOS 6.6 with Linux kernel v0292 2.6.32-431.29.2.el6.x86_64. The reference sequence C34chromosome version 3 was indexed with “bowtie2-build” and paired-end reads aligned with “bowtie2” algorithms, both using default parameters.

The alignment output in SAM format was converted to BAM format, sorted and indexed with SAMtools [18] version 0.1.18 (r982:295) (Windows 64bits binary from <https://bow.codeplex.com/releases/view/87911> and run as BWA). SAMtools “mpileup” algorithm was used to calculate genotype likelihoods at each position of the alignment. The BCF file generated by “mpileup” was processed with bcftools version 0.1.17-dev (r973:277) (Windows 64bits binary from <https://bow.codeplex.com/releases/view/87911> and run as BWA) to call potential variant SNPs and indels. The generated VFC file was visualised with an advanced text editor.

The sorted-BAM alignments were also converted to a GAP5 database with “tg_index” program included in Staden Package version 2.0.0b10 [10,11] and visually examined for insertions/omissions reported in the VFC files based on both of BWA and Bowtie 2 alignments.

The commands used were (arbitrary file names given in italics):

**BWA alignment**

bwa index *chromosome.fna*

bwa bwasw *chromosome.fna illumina_reads_pair1.fastq illumina_reads_pair2.fastq* > *alignment.sam*

**Bowtie 2 alignment**

bowtie2-build *chromosome.fna index_prefix*

bowtie2 *index_prefix* -1 *illumina_reads_pair1.fastq* -2 *illumina_reads_pair2.fastq* -S *alignment.sam*

**SAMtools processing (common to both)**

samtools view -b -S -o *alignment.bam alignment.sam*

samtools sort *alignment.bam alignment-sort*

samtools index *alignment-sort.bam*

samtools mpileup -g -f *chromosome.fna alignment-sort.bam* > *variants.bcf*

bcftools view -c -v *variants.bcf* > *variants.vcf*

tg_index -b *alignment-sort.bam*

## Annotation of genetic features in the *S. leeuwenhoekii* chromosome

Putative protein coding sequences (PCS) were called using Prodigal [20]. A FASTA formatted file containing the full *S. leeuwenhoekii* chromosome version 4 was submitted to Prodigal web server (<http://prodigal.ornl.gov/>), selecting options “Translation Table Code”=11 (Standard Bacteria/Archaea), “Treat N's as masked RNA genes”=No (Allow gene models to cross gaps), “Allow Genes to Run Off Edges”=Yes, “Output Options”= Gene Coordinates Only.

The possible functions of proteins encoded by the CDSs called by Prodigal were annotated using the BASys [14] web server (<https://www.basys.ca/>). The Prodigal web-server output was saved as a text file and processed with an advanced text editor to format a table of PCSs suitable for BASys; the following set of regular expressions was used to reformat the table (note that the PCSs are called “CDS” by Prodigal, as Genbank and EMBL formats):

To remove all lines until the one starting with “DEFINITION” (this can easily be done manually):

find: [ -~\n\r]+(^DEFINITION)

replace with: \1

To remove the last line containing “//” (this can easily be done manually):

find: ^//

replace with: (empty)

To remove spaces and CDS string and prepare for formatting a table:

find: ^\s+CDS\s+

replace with: (empty)

To prepare columns of CDS coordinates in forward strand:

find: ^(\d+)[^\d]+(\d+)

replace with: \1\t\2\t+

To prepare columns of CDS coordinates in reverse strand:

find: ^[^\d]+(\d+)[^\d]+(\d+)\)

replace with: \2\t\1\t-

To add the first header line:

find: ^DEFINITION[ -~]+

replace with: START\tEND\tSTRAND\tIDENTIFIER\tname

BASys does not require a “name” for each or any of the lines, but it does require a unique Identifier; we loaded the tab-delimited table created in a spreadsheet and generated incremental identifiers starting with “sle_00010” and increments of 10 (to provide numbers for any subsequently identified CDSs, and rRNA and tRNA genes). A FASTA formatted file containing the full *S. leeuwenhoekii* chromosome version 4 was submitted to the BASys web server selecting the options “linear contig” and “Bacterial genetic code”. Clusters of Orthologous Genes [13] were also calculated with BASys.

The chromosome sequence was also annotated using the RAST [21-23] web server (<http://rast.nmpdr.org/>) using the default options for bacterial genomes. We eventually did not use the CDS calls and annotations made by RAST but we incorporated the rRNA and tRNA annotations in the Prodigal+BASys annotated sequence to generate the published *S. leeuwenhoekii* chromosome sequence.

## Assembly and annotation of pSLE1 86.4 kb circular plasmid

BLAST analysis of the PacBio 94746 bp contig, the second largest contig, against itself revealed that the first 8.4 kb was also present as a direct repeat at the end of the contig, suggesting a circular DNA molecule. BLAST analysis of the PacBio 94746 bp contig against the Illumina contigs identified a 86370 bp contig which did not contain any repeated sequence but that contained, between position 17.6 kb and 26 kb, the full 8.4 kb segment that was repeated at the ends of the PacBio contig (Additional File 4: Figure S1).

To obtain a sequence with maximal accuracy, the precise extent of the repeated segment was identified in the PacBio and Illumina contigs by aligning about 9 kb from each end of the PacBio contig with and the equivalent Illumina sequence using ClustalX [24]. The exact extent of the repeat was identified as segments 1-8433 and 86328-94746 of the PacBio contig, and 17676-26127 of the Illumina contig. We then assembled all sequences with GAP4 of Staden Package version 2.0.0b10 [10]; we first rearranged the PacBio contig by moving the first repeat to a new contig, and the sequence from position 68657 to 94746 (including the last repeat) to the start of the contig; in doing so, we replicated the Illumina contig within the PacBio contig and both PacBio and Illumina contigs would match from position 1, facilitating the assembly in GAP4 and decision-making over a consensus sequence; the new PacBio contig, the Illumina contig, and the extracted repeat were assembled and analysed in GAP4. The PacBio and Illumina sequences were consistent for most of the alignment, disagreeing mainly in the PacBio repeats, which we interpret as errors in the PacBio sequence. All of the PacBio sequence was present in the Illumina contigs; when we identified a possible insertion or omission in the PacBio sequence, it always occurred within a homopolymeric run of Cs or Gs. After accepting the Illumina sequence as the correct one, we obtained a single consensus sequence that was 100% identical to the Illumina contig of 86370 bases.

CDSs were identified with Prodigal and the putative function manually annotated in Artemis, using BLAST and Pfam searches. For most of the CDSs, in particular those encoding putative phage particle proteins, the annotation was based on that of pZL12 [25] a well characterised plasmid with high homology and synteny with pSLE1.

## Assembly and annotation of pSLE2 >132 kb linear plasmid

A BLAST database was constructed with the “corrected reads” of the PacBio sequence (also called “Pre-assembled reads”; these are the longer or “seed” reads corrected with short high-quality circular-consensus reads that are assembled in the final scaffold prior to polishing by reassembly of all reads by the HGAP assembly pipeline [15]). Blast searches with Illumina contigs 0079 and 0026 revealed that both contigs were contiguous and some PacBio corrected-reads bridged both Illumina contigs.

A BLAST search with the first 2 kb of the 5’-end of Illumina contig 0079 against the corrected-reads database identified 47 PacBio reads, which were assembled in GAP4 of Staden Package version 2.0.0b10 [10]. Illumina contigs 0079 and 0026 were consecutively added to the assembly to obtain a final single-contig assembly of 132226 bp.

To test if the Illumina contigs had been assembled correctly, the PacBio corrected-reads database was queried with Illumina contig 0026 (obtaining 501 positive reads) and contig 0079 (obtaining 489 positive reads). All reads were carefully assembled in GAP4, using the “find internal joins” feature to merge together contigs and reads not assembled automatically; a 124467 bp consensus sequence was obtained that perfectly matched the previously obtained 132 kb sequence, providing further evidence that the sequence assembled in the Illumina contigs is correct. The PacBio data did not contain any additional sequence that could extend the Illumina contigs or identify the possible TIRs of the linear plasmid; the ends of the 132 kb sequence were not represented in the PacBio corrected-reads, hence the shorter consensus sequence.

CDSs were identified with Prodigal and the putative function manually annotated in Artemis, using BLAST and Pfam searches.

# References

1. Busarakam K, Bull AT, Girard G, Labeda DP, van Wezel GP, Goodfellow M: ***Streptomyces leeuwenhoekii* sp. nov., the producer of chaxalactins and chaxamycins, forms a distinct branch in *Streptomyces* gene trees.** *Antonie Van Leeuwenhoek* 2014, **105**(5):849-861

2. Kieser T, Bibb MJ, Buttner MJ, Chater KF, Hopwood DA: *Practical Streptomyces Genetics*. John Innes Foundation, Norwich, UK, 2000

3. Sambrook J, Fitsch EF, Maniatis T: *Molecular Cloning: A Laboratory Manual.* Cold Spring Harbor, Cold Spring Harbor Press 1989

4. Rutherford K, Parkhill J, Crook J, Horsnell T, Rice P, Rajandream MA, Barrell B: **Artemis: sequence visualization and annotation.** *Bioinformatics* 2000, **16**(10):944-945

5. Carver T, Böhme U, Otto TD, Parkhill J, Berriman M: **BamView: viewing mapped read alignment data in the context of the reference sequence.** *Bioinformatics* 2010, **26**(5):676-677

6. Carver TJ, Rutherford KM, Berriman M, Rajandream M-A, Barrell BG, Parkhill J: **ACT: the Artemis Comparison Tool.** *Bioinformatics* 2005, **21**(16):3422-3423

7. Altschul SF, Madden TL, Schäffer AA, Zhang J, Zhang Z, Miller W, Lipman DJ: **Gapped BLAST and PSI-BLAST: a new generation of protein database search programs.** *Nucleic Acids Res* 1997, **25**(17):3389-3402

8. Santiago-Sotelo P, Ramirez-Prado JH: **prfectBLAST: a platform-independent portable front end for the command terminal BLAST+ stand-alone suite.** *Biotechniques* 2012, **53**(5):299-300

9. Finn RD, Bateman A, Clements J, Coggill P, Eberhardt RY, Eddy S R, Heger A, Hetherington K, Holm L, Mistry J, Sonnhammer ELL, Tate J, Punta M: **Pfam: the protein families database.** *Nucleic Acids Res* 2014, **42**(Database issue):D222-D230.

10. Staden R, Beal KF, Bonfield JK: **The Staden Package, 1998.** In *Bioinformatics Methods and Protocols.* Edited by Misener S, Stephen A Krawetz SA. Humana Press, 1999, 115-130

11. Bonfield JK, Whitwham A: **Gap5–editing the billion fragment sequence assembly.** *Bioinformatics* 2010, **26**(14):1699-1703

12. Blin K, Medema MH, Kazempour D, Fischbach MA, Breitling R, Takano E, Weber T: **antiSMASH 2.0–a versatile platform for genome mining of secondary metabolite producers.** *Nucleic Acids Res* 2013, **41**(W1):W204-W212

13. Tatusov RL, Koonin EV, Lipman DJ: **A genomic perspective on protein families.** *Science* 1997, **278**(5338):631-637

14. Van Domselaar GH, Stothard P, Shrivastava S, Cruz JA, Guo A, Dong X, Lu P, Szafron D, Greiner R, Wishart DS: **BASys: a web server for automated bacterial genome annotation.** *Nucleic Acids Res* 2005, **33**(Web Server issue):W455-W459

15. Chin C-S, Alexander DH, Marks P, Klammer AA, Drake J, Heiner C, Clum A, Copeland A, Huddleston J, Eichler EE, Turner SW, Korlach J: **Nonhybrid, finished microbial genome assemblies from long-read SMRT sequencing data.** *Nat Methods* 2013, **10**(6):563-569

16. Li H, Durbin R: **Fast and accurate short read alignment with Burrows-Wheeler transform.** *Bioinformatics* 2009, **25**(14):1754-1760

17. Li , Durbin R: **Fast and accurate long-read alignment with Burrows-Wheeler transform.** *Bioinformatics* 2010, **26**(5):589-595

18. Li H, Handsaker B, Wysoker A, Fennell T, Ruan J, Homer N, Marth G, Abecasis G, Durbin R, 1000 Genome Project Data Processing Subgroup: **The Sequence Alignment/Map format and SAMtools.** *Bioinformatics* 2009, **25**(16):2078-2079

19. Langmead B , Salzberg SL: **Fast gapped-read alignment with Bowtie 2.** *Nat Methods* 2012, **9**(4):357-359

20. Hyatt D, Chen G-L, Locascio PF, Land ML, Larimer FW, Hauser LJ: **Prodigal: prokaryotic gene recognition and translation initiation site identification**. *BMC Bioinformatics* 2010, **11**:119

21. Aziz RK, Bartels D, Best AA, DeJongh M, Disz T, Edwards RA, Formsma K, Gerdes S, Glass EM, Kubal M, Meyer F, Olsen GJ, Olson R, Osterman AL, Overbeek RA, McNeil LK, Paarmann D, Paczian T, Parrello B, Pusch GD, Reich C, Stevens R, Vassieva O, Vonstein V, Wilke A, Zagnitko O: **The RAST Server: rapid annotations using subsystems technology.** *BMC Genomics* 2008, **9**:75

23. Overbeek R, Olson R, Pusch GD, Olsen GJ, Davis JJ, Disz T, Edwards RA, Gerdes S, Parrello B, Shukla M, Vonstein V, Wattam AR, Xia F, Stevens R: **The SEED and the Rapid Annotation of microbial genomes using Subsystems Technology (RAST).** *Nucleic Acids Res* 2014, **42**(Database issue):D206-D214

24. Thompson JD, Gibson TJ, Plewniak F, Jeanmougin F, Higgins DG: **The CLUSTAL_X windows interface: flexible strategies for multiple sequence alignment aided by quality analysis tools.** *Nucleic Acids Res* 1997, **25**(24):4876-4882

25. Zhong L, Cheng Q, Tian X, Zhao L, Qin Z: **Characterization of the replication, transfer, and plasmid/lytic phage cycle of the *Streptomyces* plasmid-phage pZL12.** *J Bacteriol* 2010, **192**(14):3747-3754
