# Supplementary material for: The Streptomyces leeuwenhoekii genome: de novo sequencing and assembly in single contigs of the chromosome, circular plasmid pSLE1 and linear plasmid pSLE2
Source: BMC Genomics. 2015 Jun 30;16(1):485. doi: 10.1186/s12864-015-1652-8 (PMC4487206; doi:10.1186/s12864-015-1652-8)

**The *Streptomyces* *leeuwenhoekii* genome: *de novo* sequencing and assembly in single contigs of the chromosome, circular plasmid pSLE1 and linear plasmid pSLE2.**

### Juan Pablo Gomez-Escribano^1*^, Jean Franco Castro^1,2^, Valeria Razmilic^1,2^, Govind Chandra^1^, Barbara Andrews^2^, Juan A. Asenjo^2^, Mervyn J. Bibb^1^

^1^Department of Molecular Microbiology, John Innes Centre, Norwich Research Park, Norwich, NR4 7UH, United Kingdom

^2^Centre for Biotechnology and Bioengineering (CeBiB), Universidad de Chile, Beauchef 850, Santiago, Chile

## Availability of data

The fully annotated sequences presented in this work have been deposited in the European Nucleotide Archive under Study accession number PRJEB8583 (<http://www.ebi.ac.uk/ena/data/view/PRJEB8583>). Each sequence has been assigned the accession codes:

**Replicon Accession ENA_Link**

pSLE1 LN831788 <http://www.ebi.ac.uk/ena/data/view/LN831788>

pSLE2 LN831789 <http://www.ebi.ac.uk/ena/data/view/LN831789>

Chromosome LN831790 <http://www.ebi.ac.uk/ena/data/view/LN831790>

**Additional File 3:**

# Determination of the Terminal Inverted Repeats

## Additional File 3: Figure S1 - Identification of the Terminal Inverted Repeat

The top panel shows an overall view of the chromosome, the bottom two panels show expanded views of the end segments, boxed in red and blue, to facilitate interpretation of the repeated and inverted sequence (black boxes). The last 7 kb at the right end of the chromosome was found to be repeated and inverted at about 388 kb from the start of the sequence (black lines, and black boxes in the expanded views at the bottom of the figure). The TIR likely extends for over 388 kb (the orange segment from position 1).


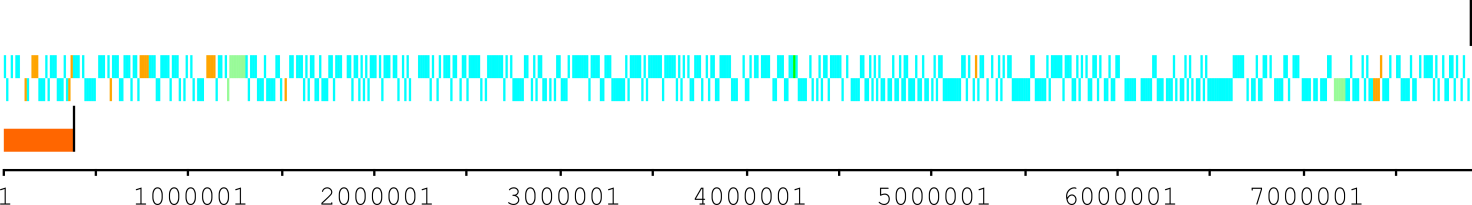


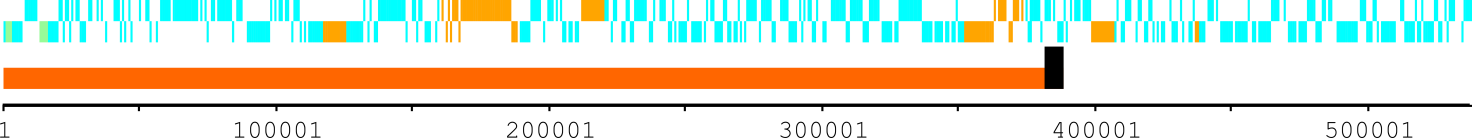


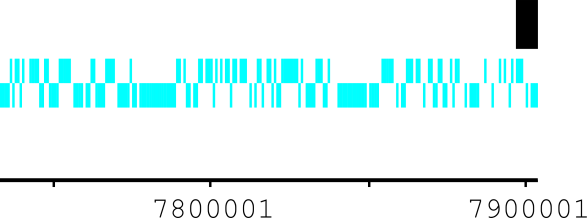


## Additional File 3: Figure S2 – Increased coverage of the Terminal Inverted Repeat

Top panel: Coverage plot of the PacBio assembly of the large 7.9 Mb contig containing an almost complete chromosome. The large blue horizontal arrow represents C34-chromosome version 4; note that the first 5 kb originates from extra sequence found only in the Illumina assembly. Bottom panel: Expanded view of the region enclosed in the red box that contains the first ~520 kb to demonstrate the increased coverage of the TIR region.


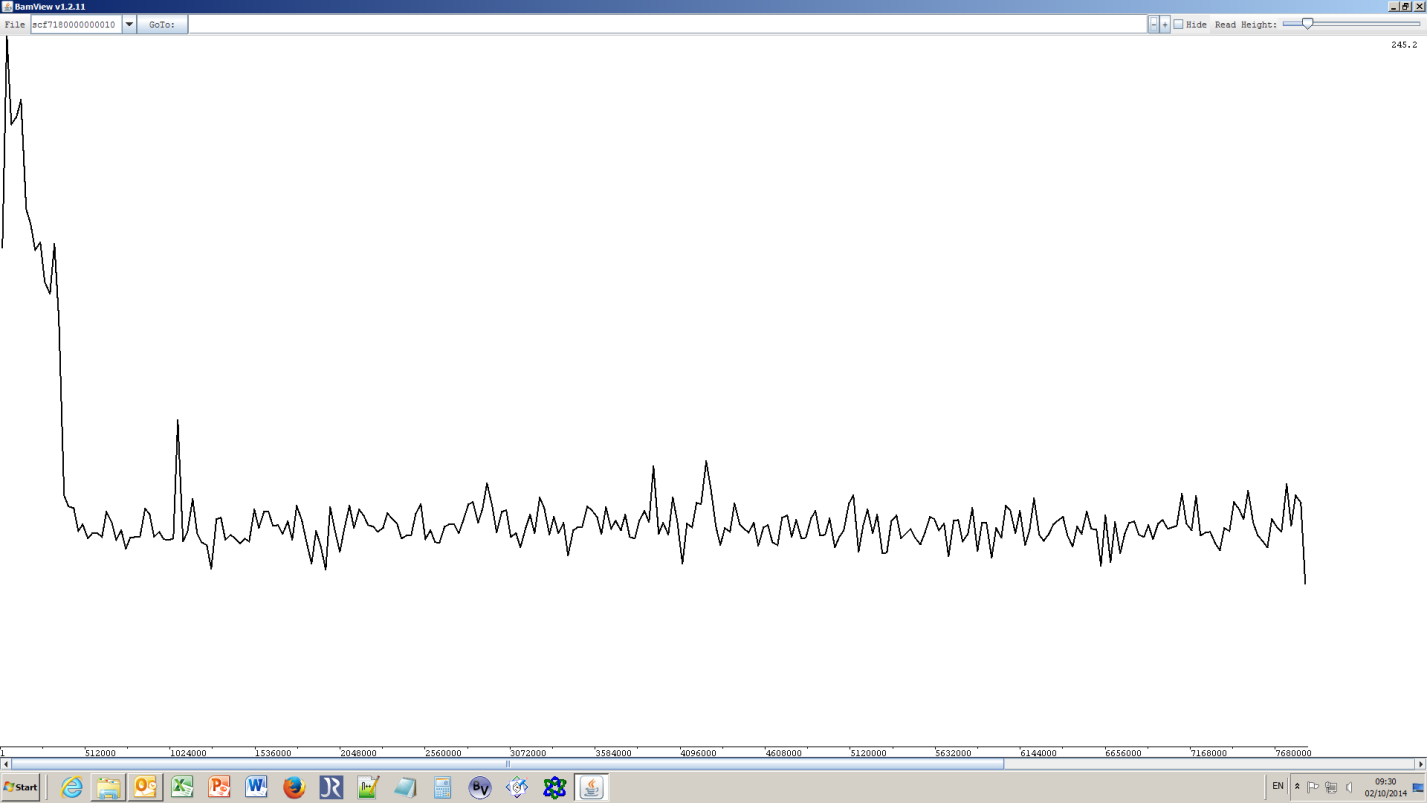


512 000 nt

5 kb added from Illumina data


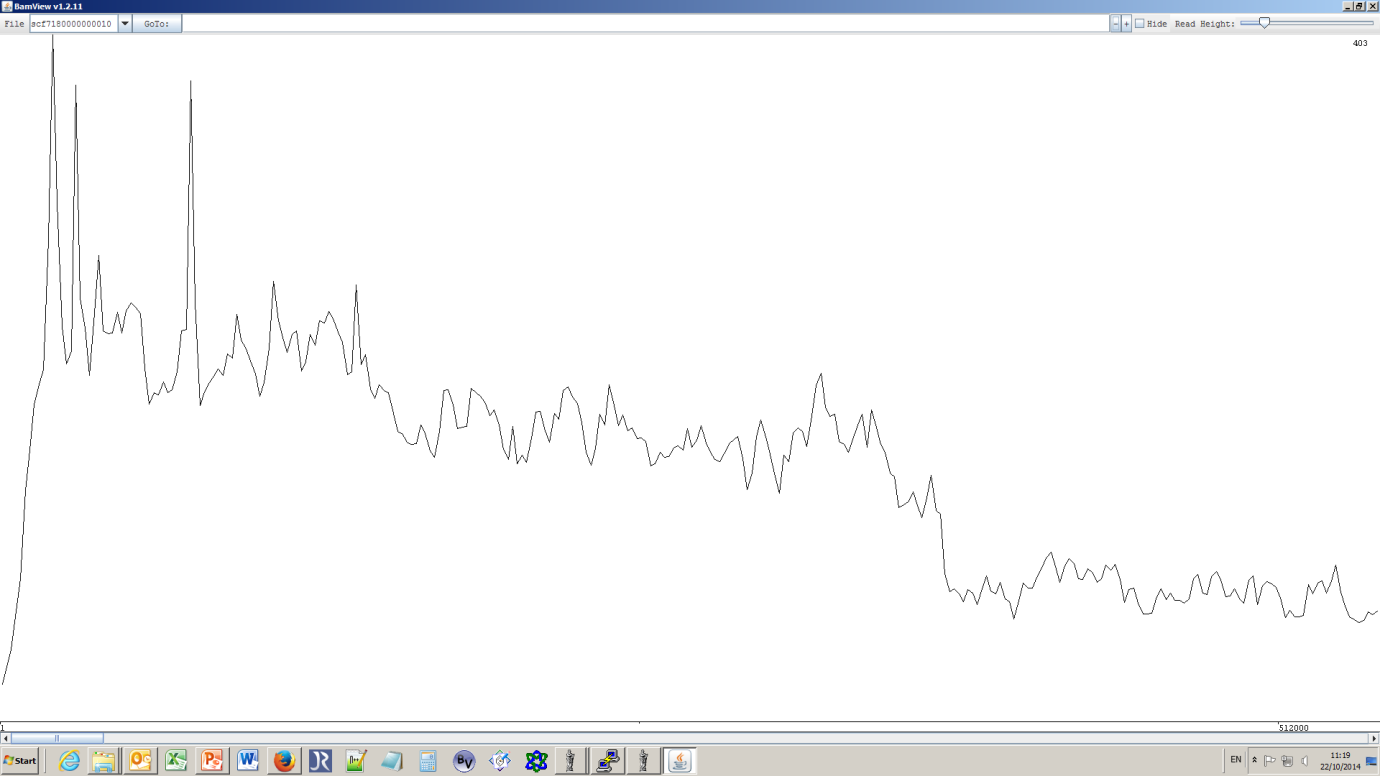


512 000 nt

256 000 nt

384 000 nt

(estimated)

## Additional File 3: Figure S3 – Genetic context at the start of the left end of the chromosome

Top image, genetic context at the start of the left end of the chromosome (TIR). The two genes encoding putative terminal helicases are highlighted and in green; only the helicase genes share high identity, the rest of the sequence is not repeated. Bottom image, the two most energetically stable potential secondary structures formed by the 1 kb upstream of *sle_00020* as predicted by Mfold; these resemble the typical predicted secondary structures found at the end of *Streptomyces* chromosomes; the sequence upstream of *sle_00120* did not show similar potential.

**
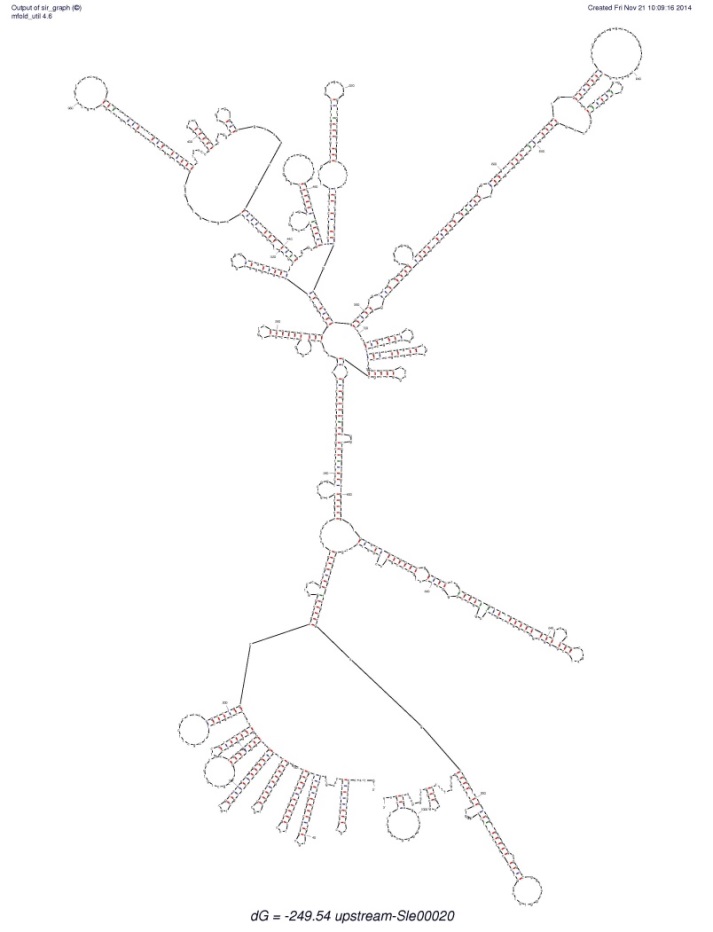

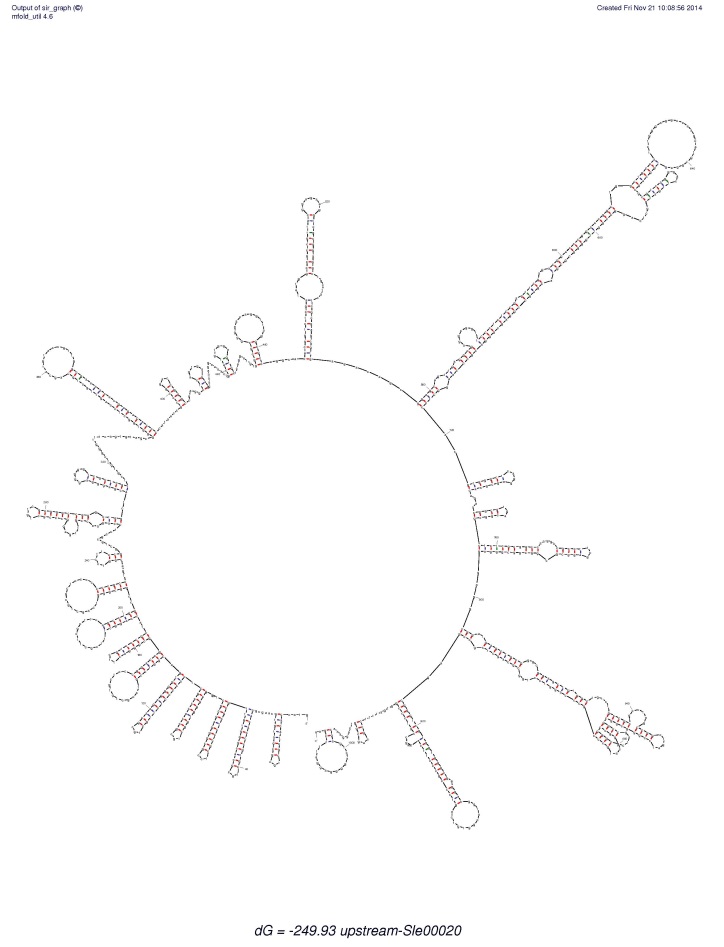
**
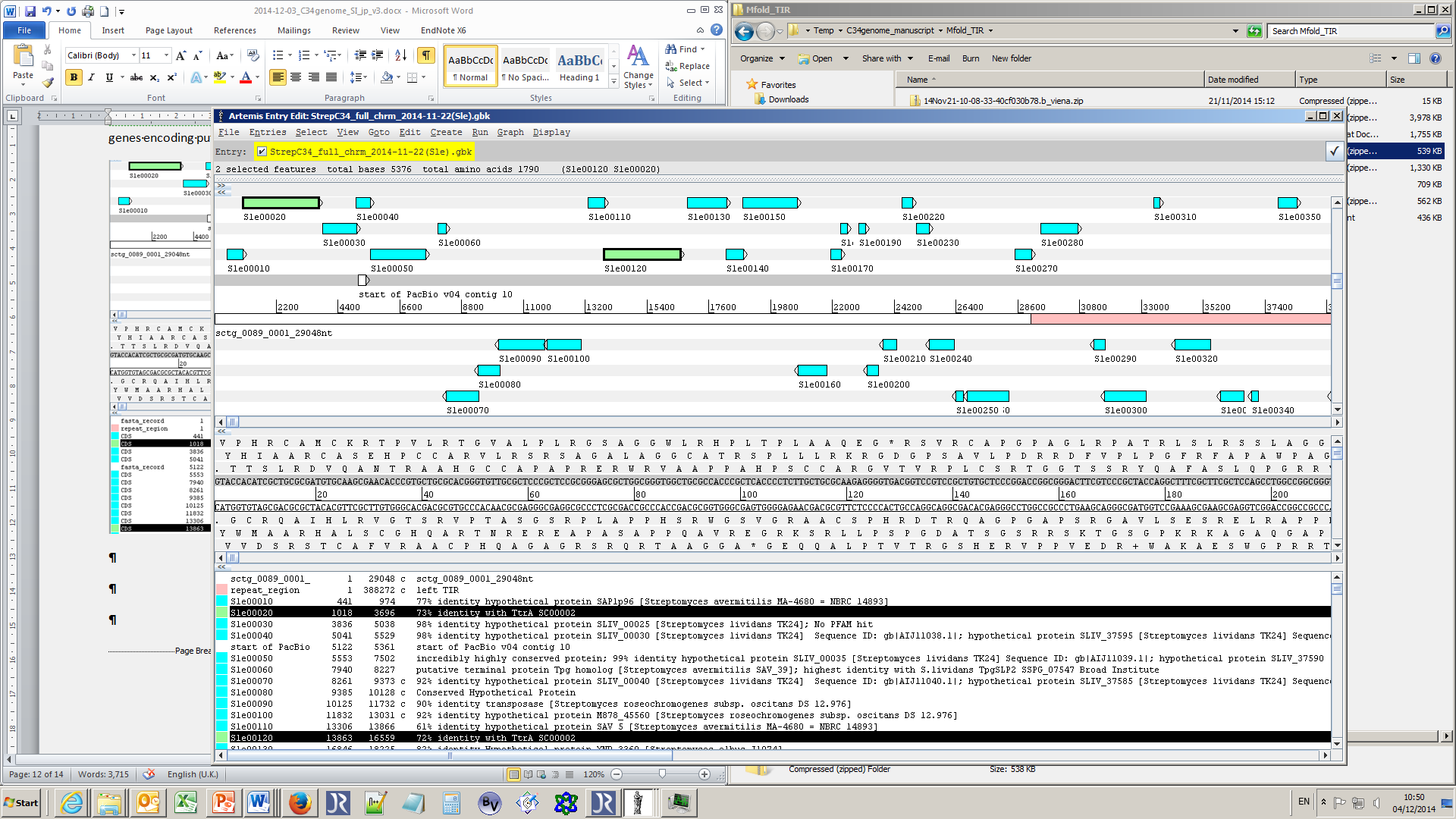

Supplement: Additional file 3: — Determination of Terminal Inverted Repeat. Three figures illustrating and supporting the identification of the Terminal Inverted Repeat. [file 12864_2015_1652_MOESM3_ESM.docx]
