# Supplementary material for: The Streptomyces leeuwenhoekii genome: de novo sequencing and assembly in single contigs of the chromosome, circular plasmid pSLE1 and linear plasmid pSLE2
Source: BMC Genomics. 2015 Jun 30;16(1):485. doi: 10.1186/s12864-015-1652-8 (PMC4487206; doi:10.1186/s12864-015-1652-8)

**The *Streptomyces* *leeuwenhoekii* genome: *de novo* sequencing and assembly in single contigs of the chromosome, circular plasmid pSLE1 and linear plasmid pSLE2.**

### Juan Pablo Gomez-Escribano^1*^, Jean Franco Castro^1,2^, Valeria Razmilic^1,2^, Govind Chandra^1^, Barbara Andrews^2^, Juan A. Asenjo^2^, Mervyn J. Bibb^1^

^1^Department of Molecular Microbiology, John Innes Centre, Norwich Research Park, Norwich, NR4 7UH, United Kingdom

^2^Centre for Biotechnology and Bioengineering (CeBiB), Universidad de Chile, Beauchef 850, Santiago, Chile

## Availability of data

The fully annotated sequences presented in this work have been deposited in the European Nucleotide Archive under Study accession number PRJEB8583 (<http://www.ebi.ac.uk/ena/data/view/PRJEB8583>). Each sequence has been assigned the accession codes:

**Replicon Accession ENA_Link**

pSLE1 LN831788 <http://www.ebi.ac.uk/ena/data/view/LN831788>

pSLE2 LN831789 <http://www.ebi.ac.uk/ena/data/view/LN831789>

Chromosome LN831790 <http://www.ebi.ac.uk/ena/data/view/LN831790>

**Additional File 4:**

# Assembly of circular plasmid pSLE1

## Additional File 4: Figure S1 – Assembly of pSLE1

Organisation of PacBio (orange) and Illumina (green) contigs for the assembly of pSEL1; in blue boxes, 8.4 kb directly repeated sequences.


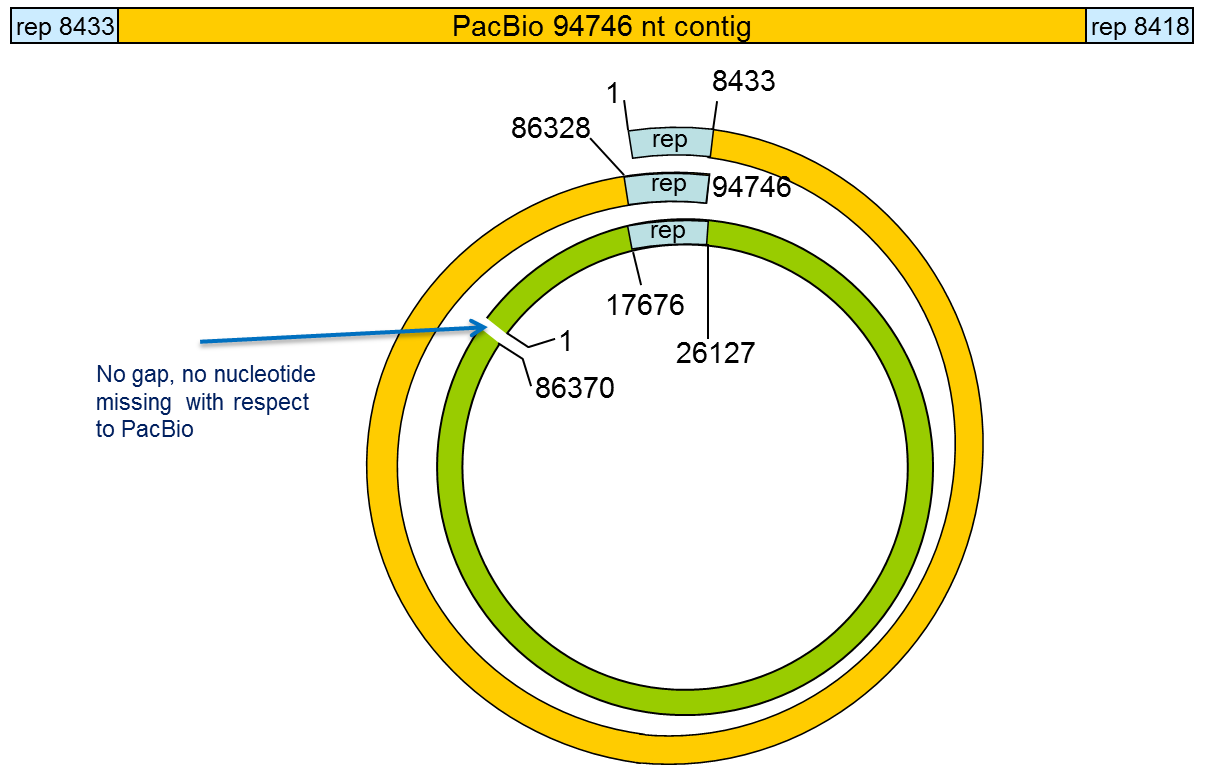

Supplement: Additional file 4: — Assembly of circular plasmid pSLE1. Figure illustrating the organisation of Illumina and PacBio data covering pSLE1. [file 12864_2015_1652_MOESM4_ESM.docx]
