# Supplementary material for: The Streptomyces leeuwenhoekii genome: de novo sequencing and assembly in single contigs of the chromosome, circular plasmid pSLE1 and linear plasmid pSLE2
Source: BMC Genomics. 2015 Jun 30;16(1):485. doi: 10.1186/s12864-015-1652-8 (PMC4487206; doi:10.1186/s12864-015-1652-8)

**The *Streptomyces* *leeuwenhoekii* genome: *de novo* sequencing and assembly in single contigs of the chromosome, circular plasmid pSLE1 and linear plasmid pSLE2.**

### Juan Pablo Gomez-Escribano^1*^, Jean Franco Castro^1,2^, Valeria Razmilic^1,2^, Govind Chandra^1^, Barbara Andrews^2^, Juan A. Asenjo^2^, Mervyn J. Bibb^1^

^1^Department of Molecular Microbiology, John Innes Centre, Norwich Research Park, Norwich, NR4 7UH, United Kingdom

^2^Centre for Biotechnology and Bioengineering (CeBiB), Universidad de Chile, Beauchef 850, Santiago, Chile

## Availability of data

The fully annotated sequences presented in this work have been deposited in the European Nucleotide Archive under Study accession number PRJEB8583 (<http://www.ebi.ac.uk/ena/data/view/PRJEB8583>). Each sequence has been assigned the accession codes:

**Replicon Accession ENA_Link**

pSLE1 LN831788 <http://www.ebi.ac.uk/ena/data/view/LN831788>

pSLE2 LN831789 <http://www.ebi.ac.uk/ena/data/view/LN831789>

Chromosome LN831790 <http://www.ebi.ac.uk/ena/data/view/LN831790>

**Additional File 5:**

# Assembly of linear plasmid pSLE2

## Additional File 5: Figure S1 – Putative pSLE2 seen in PFGE.

Pulse-Field Gel-Electrophoresis (PFGE) of total DNA isolated from *S. leeuwenhoekii* (left lane) showing the possible extrachromosomal replicon. Size marker (right lane) MidRange I PFG (New England Biolabs N3551S).


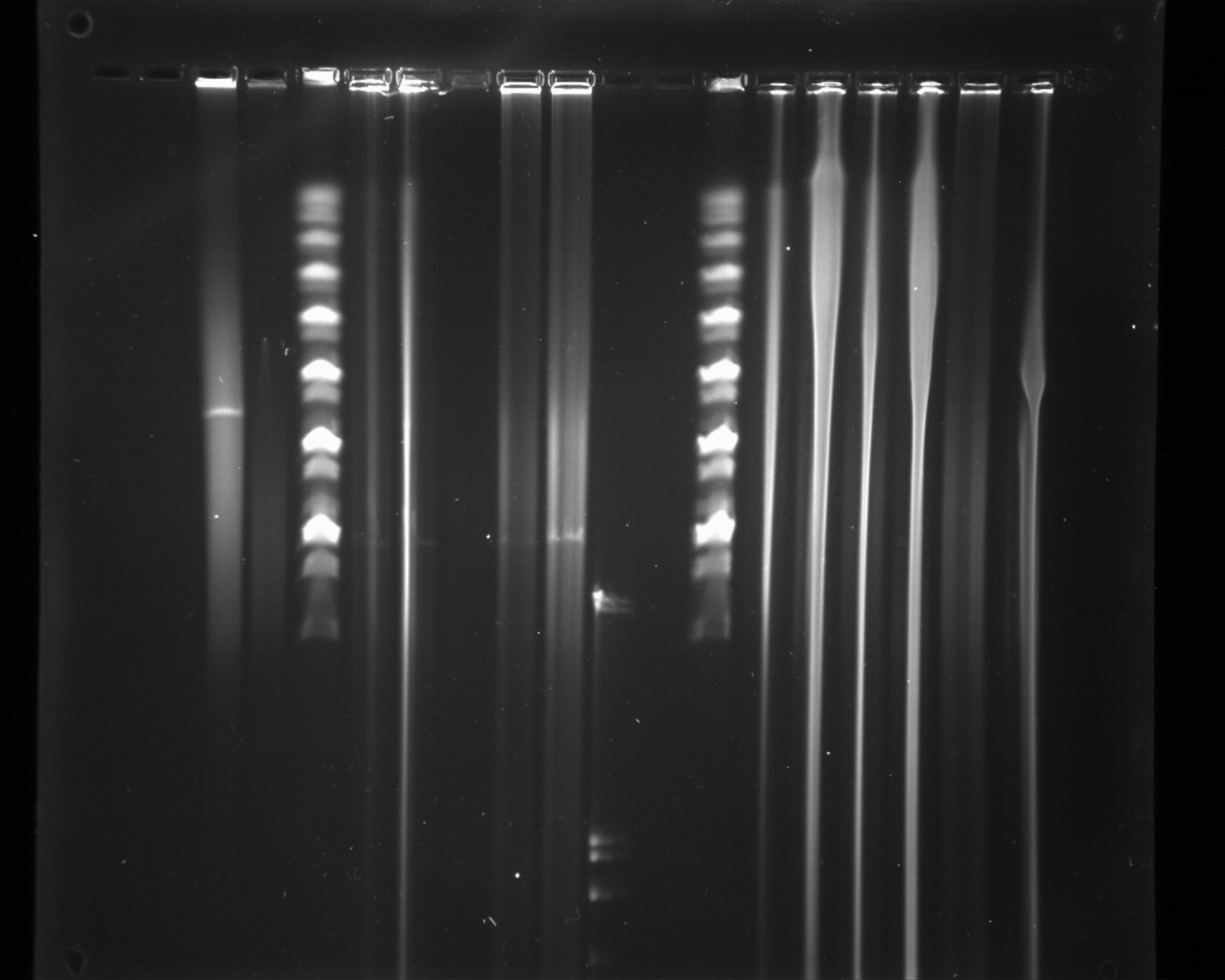

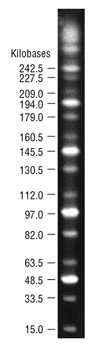


## Additional File 5: Figure S2 – Comparison of chromosome and pSLE2 left ends.

Screenshot of Artemis Comparison Tool showing the high identity shared between the ends of the chromosome (top) and pSLE2 (bottom). The red strips indicate nucleotide identity between 83% and 94%. CDSs in light green represent *ttrA* (terminal helicase) homologs.


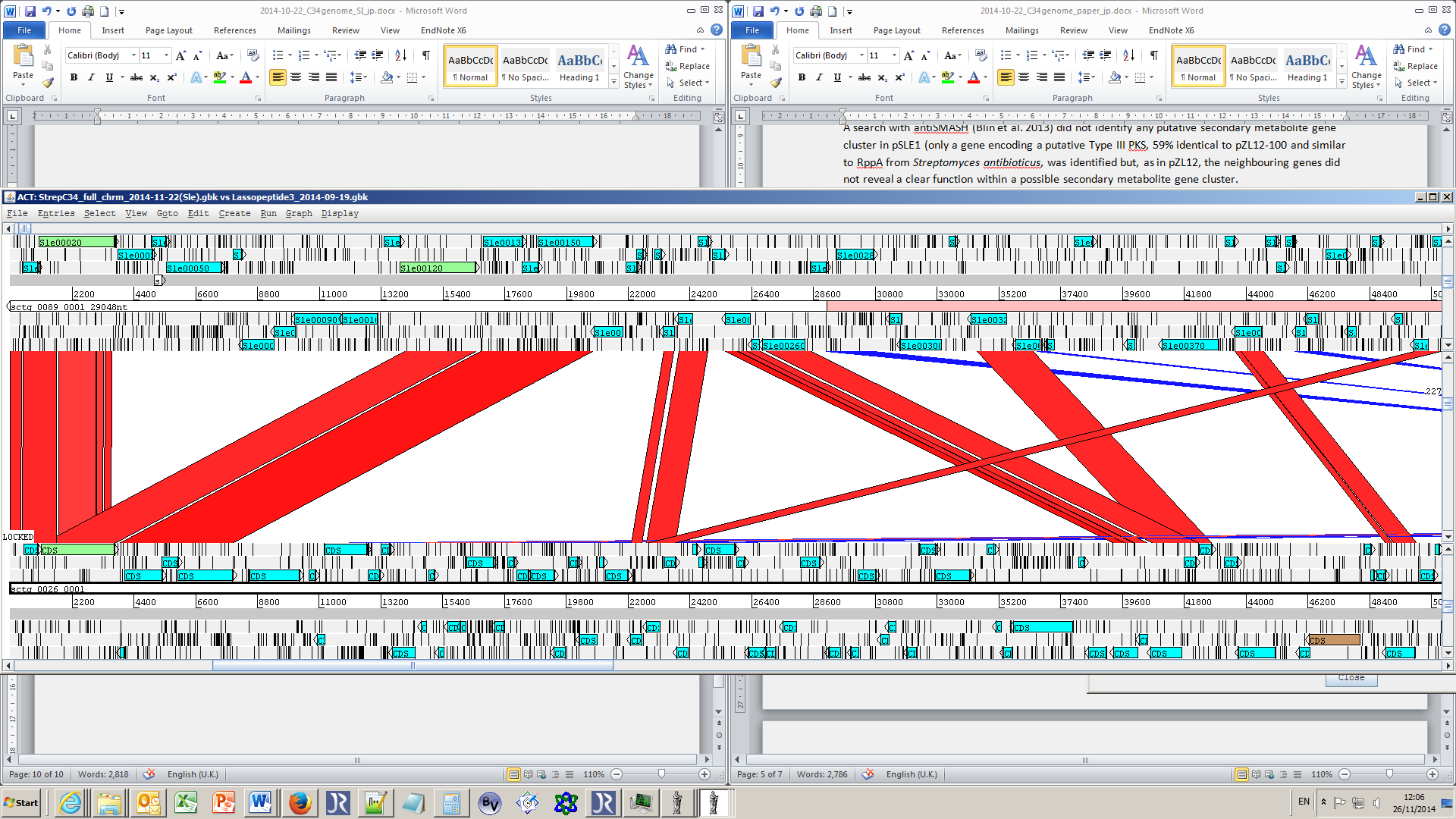

Supplement: Additional file 5: — Assembly of linear plasmid pSLE2. Two figures showing an extrachromosomal replicon, possibly pSLE2, after Pulse Field Gel Electrophoresis, and the similarity between the chromosome and pSLE2 left ends. [file 12864_2015_1652_MOESM5_ESM.docx]
