# Supplementary material for: The Streptomyces leeuwenhoekii genome: de novo sequencing and assembly in single contigs of the chromosome, circular plasmid pSLE1 and linear plasmid pSLE2
Source: BMC Genomics. 2015 Jun 30;16(1):485. doi: 10.1186/s12864-015-1652-8 (PMC4487206; doi:10.1186/s12864-015-1652-8)
Supplement: Additional file 6: — rRNA operons and tRNA genes. Two tables with the identified rRNA operons and tRNA genes. [file 12864_2015_1652_MOESM6_ESM.docx]

**The *Streptomyces* *leeuwenhoekii* genome: *de novo* sequencing and assembly in single contigs of the chromosome, circular plasmid pSLE1 and linear plasmid pSLE2.**

### Juan Pablo Gomez-Escribano^1*^, Jean Franco Castro^1,2^, Valeria Razmilic^1,2^, Govind Chandra^1^, Barbara Andrews^2^, Juan A. Asenjo^2^, Mervyn J. Bibb^1^

^1^Department of Molecular Microbiology, John Innes Centre, Norwich Research Park, Norwich, NR4 7UH, United Kingdom

^2^Centre for Biotechnology and Bioengineering (CeBiB), Universidad de Chile, Beauchef 850, Santiago, Chile

## Availability of data

The fully annotated sequences presented in this work have been deposited in the European Nucleotide Archive under Study accession number PRJEB8583 (<http://www.ebi.ac.uk/ena/data/view/PRJEB8583>). Each sequence has been assigned the accession codes:

**Replicon Accession ENA_Link**

pSLE_1 LN831788 <http://www.ebi.ac.uk/ena/data/view/LN831788>

pSLE_2 LN831789 <http://www.ebi.ac.uk/ena/data/view/LN831789>

Chromosome LN831790 <http://www.ebi.ac.uk/ena/data/view/LN831790>

**Additional File 6:**

**Tables with genetic statistics: rRNA operons and tRNA genes**

## Additional File 6: Table S1 – Location of identified rRNA operons

Location of identified rRNA operons (separated by an empty row). (^(1)^c = complementary strand)

| **Gene Identifier** | **Start position** | **End position** | **Strand^(1)^** | **Annotation** |
| --- | --- | --- | --- | --- |
| *sle_19224* | 2364598 | 2364717 | c | 5S RNA |
| *sle_19225* | 2364821 | 2367945 | c | Large Subunit Ribosomal RNA |
| *sle_19226* | 2368128 | 2369758 | c | Small Subunit Ribosomal RNA |
|  |  |  |  |  |
| *sle_31724* | 3774415 | 3774534 | c | 5S RNA |
| *sle_31725* | 3774638 | 3777761 | c | Large Subunit Ribosomal RNA |
| *sle_31726* | 3777952 | 3779582 | c | Small Subunit Ribosomal RNA |
|  |  |  |  |  |
| *sle_33305* | 3962518 | 3962637 | c | 5S RNA |
|  |  |  |  |  |
| *sle_37804* | 4473032 | 4474662 |  | Small Subunit Ribosomal RNA |
| *sle_37805* | 4474845 | 4477969 |  | Large Subunit Ribosomal RNA |
| *sle_37806* | 4478073 | 4478192 |  | 5S RNA |
|  |  |  |  |  |
| *sle_42614* | 4991245 | 4992875 |  | Small Subunit Ribosomal RNA |
| *sle_42615* | 4993058 | 4996182 |  | Large Subunit Ribosomal RNA |
| *sle_42616* | 4996286 | 4996405 |  | 5S RNA |
|  |  |  |  |  |
| *sle_53384* | 6254341 | 6255971 |  | Small Subunit Ribosomal RNA |
| *sle_53385* | 6256152 | 6259279 |  | Large Subunit Ribosomal RNA |
| *sle_53386* | 6259403 | 6259522 |  | 5S RNA |
|  |  |  |  |  |
| *sle_57074* | 6682487 | 6684117 |  | Small Subunit Ribosomal RNA |
| *sle_57075* | 6684300 | 6687424 |  | Large Subunit Ribosomal RNA |
| *sle_57076* | 6687528 | 6687647 |  | 5S RNA |

## Additional File 6: Table S2 – Location of identified tRNA genes

Location of identified tRNA genes. ^(1)^c = complementary strand)

| **Gene Identifier** | **Start position** | **End position** | **Strand^(1)^** | **Annotation** |
| --- | --- | --- | --- | --- |
| *sle_08585* | 1013851 | 1013924 | c | tRNA-Pro-GGG |
| *sle_08865* | 1044224 | 1044297 | c | tRNA-Pro-GGG |
| *sle_09695* | 1161604 | 1161677 | c | tRNA-Pro-GGG |
| *sle_11775* | 1458894 | 1458978 | c | tRNA-Leu-GAG |
| *sle_19215* | 2363647 | 2363719 | c | tRNA-Thr-GGT |
| *sle_21053* | 2588402 | 2588474 | c | tRNA-Glu-CTC |
| *sle_21054* | 2588503 | 2588574 | c | tRNA-Gln-CTG |
| *sle_21055* | 2588610 | 2588682 | c | tRNA-Glu-CTC |
| *sle_21056* | 2588712 | 2588784 | c | tRNA-Glu-CTC |
| *sle_21057* | 2588824 | 2588895 | c | tRNA-Gln-CTG |
| *sle_23105* | 2836934 | 2837005 |  | tRNA-Arg-CCG |
| *sle_25355* | 3075622 | 3075695 | c | tRNA-Met-CAT |
| *sle_25365* | 3077849 | 3077922 | c | tRNA-Met-CAT |
| *sle_29915* | 3586910 | 3586982 | c | tRNA-Trp-CCA |
| *sle_30025* | 3596507 | 3596579 | c | tRNA-Met-CAT |
| *sle_30026* | 3596629 | 3596701 | c | tRNA-Thr-GGT |
| *sle_30115* | 3605443 | 3605524 | c | tRNA-Tyr-GTA |
| *sle_32565* | 3873094 | 3873167 | c | tRNA-Thr-CGT |
| *sle_33005* | 3929435 | 3929508 |  | tRNA-Pro-CGG |
| *sle_34215* | 4059778 | 4059848 |  | tRNA-Gly-CCC |
| *sle_35655* | 4223780 | 4223863 | c | tRNA-Leu-CAG |
| *sle_35815* | 4240142 | 4240214 | c | tRNA-Ala-TGC |
| *sle_35885* | 4246723 | 4246796 | c | tRNA-Ile-GAT |
| *sle_36825* | 4358611 | 4358695 |  | tRNA-Ser-TGA |
| *sle_36925* | 4372496 | 4372586 |  | tRNA-Ser-GCT |
| *sle_36926* | 4372860 | 4372932 |  | tRNA-Arg-ACG |
| *sle_37155* | 4395398 | 4395482 | c | tRNA-Ser-CGA |
| *sle_37345* | 4409856 | 4409940 | c | tRNA-Ser-GGA |
| *sle_37405* | 4419475 | 4419547 | c | tRNA-Gly-GCC |
| *sle_37455* | 4423276 | 4423348 | c | tRNA-Gly-GCC |
| *sle_37585* | 4436702 | 4436776 | c | tRNA-Asp-GTC |
| *sle_37604* | 4442047 | 4442120 | c | tRNA-Phe-GAA |
| *sle_37605* | 4442145 | 4442219 | c | tRNA-Asp-GTC |
| *sle_37606* | 4442269 | 4442341 | c | tRNA-Glu-TTC |
| *sle_37655* | 4449389 | 4449461 |  | tRNA-Lys-TTT |
| *sle_37665* | 4450809 | 4450882 |  | tRNA-Met-CAT |
| *sle_38865* | 4579364 | 4579436 |  | tRNA-Thr-TGT |
| *sle_40745* | 4771396 | 4771468 | c | tRNA-Arg-CCT |
| *sle_41075* | 4808641 | 4808714 |  | tRNA-Ala-CGC |
| *sle_41585* | 4866281 | 4866357 | c | tRNA-Gln-TTG |
| *sle_41895-bldA* | 4909651 | 4909734 |  | tRNA-Leu-TAA |
| *sle_43355* | 5097904 | 5097975 | c | tRNA-Cys-GCA |
| *sle_43695* | 5132561 | 5132635 | c | tRNA-Leu-TAG |
| *sle_44145* | 5190011 | 5190084 | c | tRNA-Lys-CTT |
| *sle_44175* | 5193482 | 5193555 | c | tRNA-Lys-CTT |
| *sle_44265* | 5204149 | 5204222 | c | tRNA-Lys-CTT |
| *sle_44465* | 5228148 | 5228220 | c | tRNA-His-GTG |
| *sle_45445* | 5342319 | 5342391 | c | tRNA-Arg-TCT |
| *sle_46175* | 5432933 | 5433003 | c | tRNA-Gly-TCC |
| *sle_46176* | 5433190 | 5433263 |  | tRNA-Pro-TGG |
| *sle_46595* | 5486514 | 5486586 |  | tRNA-Ala-GGC |
| *sle_46615* | 5487468 | 5487540 |  | tRNA-Ala-GGC |
| *sle_47494* | 5595605 | 5595677 |  | tRNA-Asn-GTT |
| *sle_47495* | 5595683 | 5595755 |  | tRNA-Asn-GTT |
| *sle_47496* | 5595989 | 5596062 |  | tRNA-Met-CAT |
| *sle_48395* | 5694626 | 5694697 |  | tRNA-Val-TAC |
| *sle_51255* | 6020143 | 6020215 | c | tRNA-Leu-CAA |
| *sle_54415* | 6373758 | 6373842 | c | tRNA-Leu-GAG |
| *sle_55613* | 6512130 | 6512201 | c | tRNA-Val-GAC |
| *sle_55614* | 6512242 | 6512313 | c | tRNA-Val-GAC |
| *sle_55615* | 6512334 | 6512405 | c | tRNA-Val-GAC |
| *sle_55616* | 6512410 | 6512480 | c | tRNA-Cys-GCA |
| *sle_55617* | 6512518 | 6512590 | c | tRNA-Gly-GCC |
| *sle_55705* | 6520410 | 6520481 |  | tRNA-Val-CAC |
| *sle_55765* | 6528170 | 6528241 |  | tRNA-Val-CAC |
